# Nonsense-mediated mRNA decay inhibition synergizes with MDM2 inhibition to suppress *TP53* wild-type cancer cells in p53 isoform-dependent manner

**DOI:** 10.1038/s41420-022-01190-3

**Published:** 2022-09-30

**Authors:** Ying Li, Meng Wu, Lili Zhang, Li Wan, Hexin Li, Lanxin Zhang, Gaoyuan Sun, Wei Huang, Junhua Zhang, Fei Su, Min Tang, Fei Xiao

**Affiliations:** 1 Clinical Biobank, Beijing Hospital, National Center of Gerontology, Institute of Geriatric Medicine, Chinese Academy of Medical Sciences, 100730 Beijing, P. R. China; 2 Graduate School of Peking Union Medical College, 100730 Beijing, P. R. China; 3 The Key Laboratory of Geriatrics, Beijing Institute of Geriatrics, Beijing Hospital, National Center of Gerontology, National Health Commission; Institute of Geriatric Medicine, Chinese Academy of Medical Sciences, 100730 Beijing, P. R. China; 4 Department of Urology, Beijing Hospital, National Center of Gerontology, Institute of Geriatric Medicine, Chinese Academy of Medical Sciences, 100730 Beijing, P. R. China; 5 Department of Oncology, Beijing Hospital, National Center of Gerontology, Institute of Geriatric Medicine, Chinese Academy of Medical Sciences, 100730 Beijing, P. R. China

**Keywords:** Targeted therapies, Apoptosis

## Abstract

The restoration of the normal function of the tumour suppressors, such as p53, is an important strategy in tumour therapeutics. Nonsense-mediated mRNA decay (NMD) inhibition by NMD inhibitor (NMDi) upregulates functional p53 isoforms, p53β and p53γ, and activates the p53 pathway. XR-2, a novel mouse double minute 2 homolog (MDM2) inhibitor, can disrupt the interaction between p53 and MDM2, thus decreasing the MDM2-mediated degradation of p53 and increasing the p53 protein levels. However, the combined effects of these two agents have not been thoroughly explored. This study combined XR-2 and NMDi in four *TP53* wild-types and four *TP53*-mutated cancer cell lines. The combination of these two agents achieved significant synergistic effects on *TP53* wild-type cancer cell lines by transactivating p53 target genes, inducing apoptosis, cell-cycle arrest and DNA damage repair. The p53β isoform induced by NMDi enhances the transactivation ability of p53α induced by XR-2, which partially explains the mechanism of the synergistic effects of XR-2 and NMDi. This study identified a combination treatment of NMDi and XR-2 which could serve as a novel cancer therapeutic approach for MDM2-overexpressed *TP53* wild-type cancers and delineated a future therapy based on the further reactivation of p53.

## Introduction

For efficient, stable and error-free flow of genetic information, many sophisticated mechanisms exist at each step to maintain genomics fidelity and cellular homeostasis [1–3]. An example of this mechanism is nonsense-mediated mRNA decay (NMD), one of the most important post-transcriptional mechanisms widely involved in biological and pathological processes [4]. It mainly degrades the mRNA containing premature stop codon (PTC) to avoid the formation of C-terminal truncated proteins [5]. NMD plays an important role in tumorigenesis and tumour development. On the one hand, NMD inhibits tumorigenesis by removing the mRNA that produces dominant negative-effect proteins [6, 7]. On the other hand, NMD degrades mRNA that can generate partially or fully functional tumour suppressors [8]. For example, NMD-sensitive mutation in *CDH1* mRNA is degraded by NMD [9]. NMD also degrades functional p53β/γ isoforms that are formed by alternative splicing (AS) of the *TP53* [10, 11].

The tumour suppressor p53, encoded by the *TP53* gene, can bind to specific DNA sequences and activate their transcription. It plays a role in cell apoptosis, cell-cycle progression, DNA damage and repair, ageing, metabolism, etc. [12]. The p53 protein has 12 isoforms and can be divided into p53α, p53β and p53γ three categories. p53β and p53γ are generated by AS of intron 9 of p53 pre-mRNA, which introduces a PTC and results in C-terminal truncated proteins at amino acids 341 and 345 [13, 14]. The C-terminus of full-length p53 has a negative regulatory region, under the negative regulation by E3 ubiquitin-protein ligase MDM2 (mouse double minute 2 homolog) [15]. p53β and p53γ are less susceptible to MDM2-mediated degradation as they lack ubiquitination sites in the C-terminus [16]. However, p53β and p53γ have intact DNA-binding and transcription-activating domains. As a result, they function partially as tumour suppressors like p53α isoform [13].

Tumours that express p53β/γ have a better prognosis than tumours that do not express p53β/γ [17, 18], emphasizing the biological significance of these two isoforms. The mRNA of p53β and p53γ have PTC, which makes them potential substrates of NMD, thus regulating the expression of p53β and p53γ [19, 20]. NMD inhibition significantly increased the mRNA and protein levels of p53β and p53γ, and activated the transcription of p53 target genes [11]. Thus, inducing the expressions of p53β and p53γ via NMD inhibition could be a promising approach to restoring p53 function in tumours.

In MDM2-overexpressed cancer cells, MDM2-mediated degradation of p53 is the leading cause of p53 dysfunction [21]. In this case, small molecular inhibitors that block the binding of MDM2 to p53, such as nutlin-3, RG7388, and the novel inhibitor XR-2 reported by Wu et al. has been developed [22, 23]. These MDM2 inhibitors have demonstrated a promising anti-tumour effect on *TP53* wild-type cancers. MDM2 and NMD inhibitors upregulate p53α and p53β/γ, respectively. Moreover, p53β modulates p53α transactivation ability in a promoter-specific manner [24]. However, the combined use of these two agents in the treatment and their synergistic effects are yet unclear. Therefore, this study demonstrates that combining a novel MDM2 inhibitor XR-2 [22] and NMD inhibitor (NMDi) could activate p53α and p53β, respectively, and have significant synergistic effects on *TP53* wild-type cancer cells. We further elucidated the mechanism underlying the synergistic anti-tumour effects of these two agents.

## Results

### NMD inhibition activates the p53 pathway and suppresses cancer cell viability

**Fig. 1:**
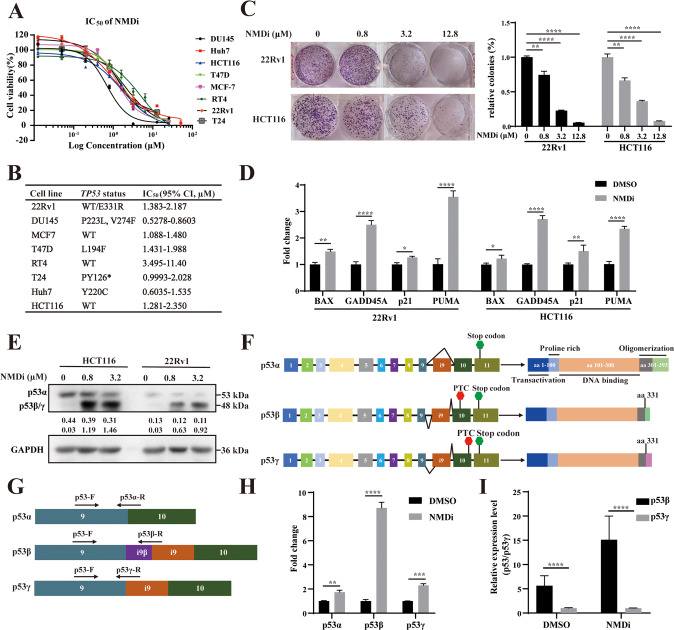
NMDi as a potential anti-cancer agent via the p53 pathway. **A** Eight solid tumour cell lines were treated with different concentrations of NMDi for 72 h, and the cell viability was measured using CCK-8 assay. **B** IC_50_ and *TP53* status of eight cell lines. Asterisk represents a nonsense mutation. **C** Images and quantitative analysis of the colony-forming assay of 22Rv1 and HCT116 cells treated with the indicated concentrations of NMDi for 24 h and cultured for 14 days. **D** mRNA expression fold change of p53 target genes (*P21*, *PUMA*, *BAX* and *GADD45A*) in 22Rv1 and HCT116 cells treated with 3.2 µM NMDi for 24 h using qPCR. **E** 22Rv1 and HCT116 cells treated with the indicated concentrations of NMDi introduced a band of p53 around 48 kDa using Western blot. **F** Schematic of three major isoforms of p53, i.e. p53α, p53β and p53γ, resulting from alternative splicing of intron 9. The green stop sign represents a normal stop codon, and the red stop sign represents a PTC. **G** Design of isoform-specific primers of the three p53 isoforms. These three isoforms share a common forward primer, and have specific reverse primers that across the splicing site. **H** Fold change in the mRNA expression level of three p53 isoforms in 22Rv1 cells treated with DMSO or 3.2 µM NMDi for 24 h using qPCR. **I** Relative fold change of p53β to p53γ in 22Rv1 cells treated with DMSO or 3.2 µM NMDi for 24 h. (Data are mean ± standard deviation, and *p*-values were calculated by ungrouped *t*-test. *p ≤ 0.05, **p ≤ 0.01, ***p ≤ 0.001, ****p ≤ 0.0001 vs. control group; ns not significant. *N* = 3).

As reported by Cheruiyot et al. [25], a small molecule inhibitor that targets SMG1, which activates the NMD pathway by phosphorylating UPF1, was used to investigate the effect of NMD inhibition as a potential approach in cancer treatment. The in vitro antiproliferative activity of NMDi in eight cell lines (DU145, Huh7, MCF7, T47D, HCT116, 22Rv1, RT4 and T24) was evaluated (Fig. 1A). IC_50_ values of NMDi and *TP53* status in eight cell lines were shown in Fig. 1B. The IC_50_ values ranged from 0.688 to 5.09 µM, and no difference was observed between different *TP53* status. The effects of NMDi on colony-forming ability were evaluated. 22Rv1 and HCT116 cell lines were selected as representatives (prostate cancer and colorectal cancer cell lines) for the following experiments. The results showed that NMDi significantly reduced the colony-forming abilities of 22Rv1 and HCT116 cells in a dose-dependent manner (*p* ≤ 0.05; Fig. 1C). To explore the relationship between the antiproliferative activity of NMDi and the p53 pathway activation, the expression of several well-known p53 target genes were measured. 22Rv1 or HCT116 cells treated with NMDi significantly increased the cell-cycle-related gene *P21* (22Rv1: *p* ≤ 0.05; HCT116: *p* ≤ 0.001) and *GADD45A* (*p* ≤ 0.0001 for both cell lines) expression compared to DMSO-treated cells (Fig. 1D). NMDi treatment also increased the expression of BAX (22Rv1: *p* ≤ 0.001; HCT116: *p* ≤ 0.05) and PUMA (*p* ≤ 0.0001 for both cell lines, Fig. 1D), which were important apoptosis-related genes. These results suggested that NMDi could serve as a potential anti-cancer agent by activating the p53 pathway.

NMD inhibition was reported to stabilize p53 isoforms. A p53 antibody that can recognize all p53 isoforms was used to determine the expression of the p53 isoforms after NMDi treatment using Western blot. As shown in Fig. 1E, NMDi did not significantly alter the expression of p53α protein in 22Rv1 and HCT116 cells. However, a band of approximately 48 kDa was detected, corresponding to the predicted size of p53β or p53γ (Fig. 1F). To further explore the extra band, isoform-specific primers were designed to detect p53 isoforms (Fig. 1G). As shown in Fig. 1H, the expression levels of p53β and p53γ were significantly induced by NMDi (*p* ≤ 0.001; Fig. 1H). However, the expression of p53γ was lower than that p53β after NMDi treatment, as the expression level of p53β was ten times higher than p53γ (*p* ≤ 0.001; Fig. 1I). These qPCR and Western blot results suggest that p53β and p53γ were sensitive to NMD, and NMDi mainly increases the expression of p53β.

### NMD and MDM2 inhibition activate the p53 pathway via different p53 isoforms

**Fig. 2:**
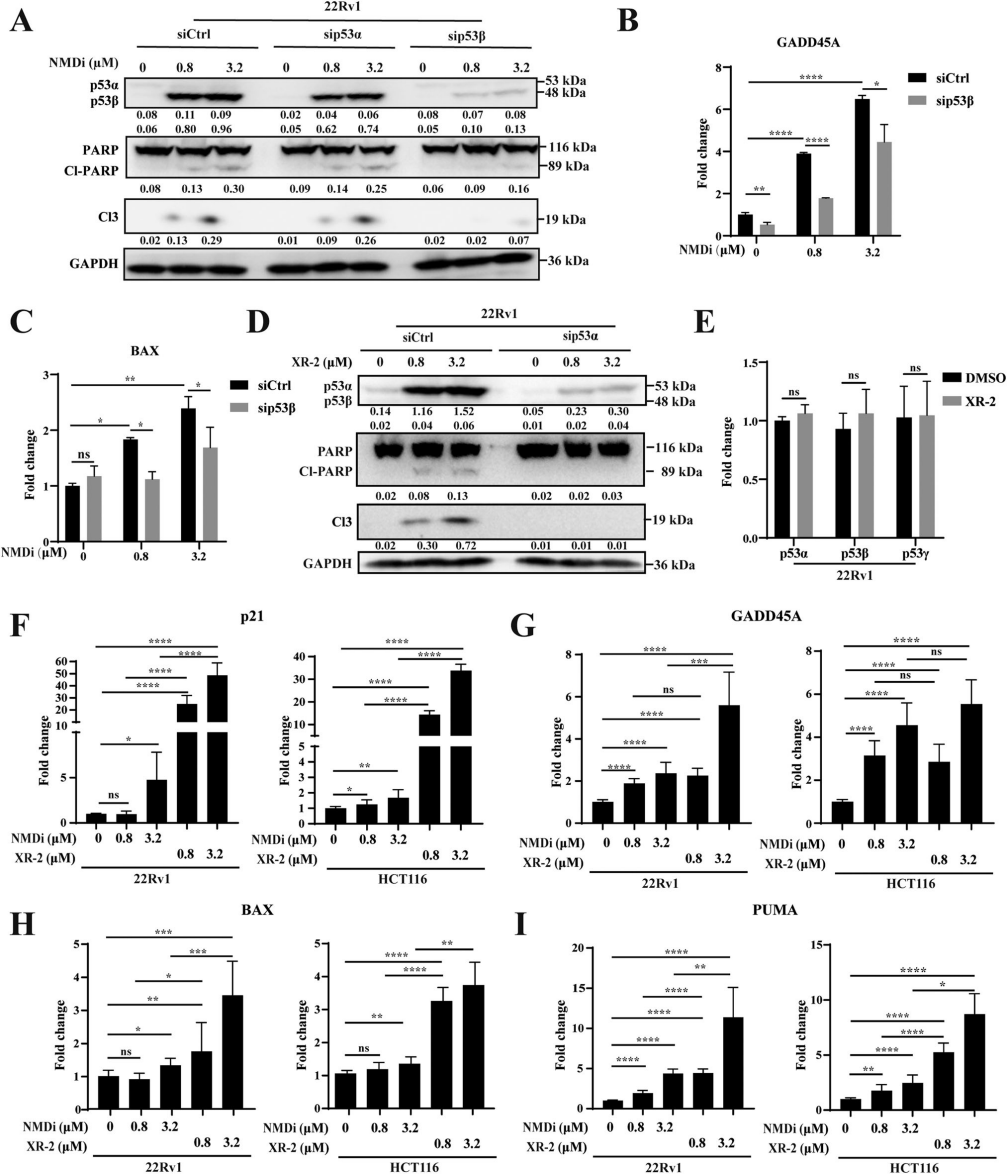
NMDi and XR-2 activated the p53 pathway. **A** Western blot analysis of 22Rv1 cells treated with siCtrl, sip53α or p53β, followed by incubation with indicated concentrations of NMDi for 48 h. **B**, **C** *BAX* and *GADD45* quantification in 22Rv1 cells treated with siCtrl or sip53β and indicated concentration of NMDi for 48 h using qPCR. **D** Western blot results of the apoptosis biomarker (cleaved PARP and cleaved Caspase 3) and p53 isoform of 22Rv1 cells treated with the indicated concentrations of XR-2 for 48 h. **E** mRNA expression level of three p53 isoforms in 22Rv1 cells treated with 3.2 µM XR-2 for 24 h using qPCR**. F**–**I** The mRNA expression fold change of key p53 target genes (*P21*, *PUMA*, *BAX* and *GADD45A*) in 22Rv1 and HCT116 cells treated with the indicated concentrations of NMDi or XR-2 for 24 h by qPCR. Cl-PARP, cleaved PARP; Cl3, cleaved Caspase 3. (Data are mean ± standard deviation, and *p*-values were calculated by the ungrouped *t*-test. *p ≤ 0.05, **p ≤ 0.01, ***p ≤ 0.001, ****p ≤ 0.0001; ns not significant. *N* = 3).

Treatment with NMDi for 48 h induced apoptosis in cells (Fig. 2A), which was confirmed by the increase in the protein levels of the apoptosis markers (cleaved PARP and cleaved Caspase 3) in 22Rv1 cells. To investigate whether NMDi induced apoptosis via p53 isoforms, the expression of p53 isoforms was decreased by siRNA. sip53β treatment significantly decreased the induced band around 48 kDa and partially rescued the NMDi-induced apoptosis (Fig. 2A), thus confirming that p53β partially mediated the apoptotic effect of NMDi. A decrease in the expression of *BAX* (*p* < 0.05) and *GADD45A* (*p* < 0.0001) was also observed on NMDi treatment (Fig. 2B, C) when knockdown p53β. These results suggest that p53β partially regulates the expression of p53 target genes.

The sensitivity of MDM2-mediated degradation of p53β and p53γ was also studied. 22Rv1 cells were treated with siControl or sip53α and different concentrations of XR-2. As depicted in Fig. 2D, XR-2 treatment increased p53α and apoptosis markers expression dose-dependently. However, the mRNA and protein expression of p53β and p53γ were not altered (Fig. 2E). Treated with sip53α significantly decreased p53α expression and apoptosis markers, thereby rescuing the XR-2 induced cell apoptosis (Fig. 2D).

The p53α and p53β contain an intact transactivation domain. Then, qPCR was performed to evaluate the transcriptional activity of p53 isoforms after XR-2 and NMDi treatment. 22Rv1 and HCT116 cells treated with XR-2 or NMDi induced the mRNA expression of p53 target genes *P21* (*p* < 0.0001), *GADD45A* (*p* < 0.0001), *PUMA* (22Rv1: *p* < 0.05 and HCT116: *p* < 0.0001) and *BAX* (*p* < 0.0001) in a dose-dependent manner compared to untreated cells (Fig. 2F–I). Thus, the XR-2 or NMDi treatment influences cell-cycle progression and apoptosis. In addition, XR-2-induced p53α expression showed much higher transactivation activity than NMDi-induced p53β. Collectively, XR-2 and NMDi activated p53α and p53β, respectively, which activated p53 pathway targeted genes and further caused cell-cycle arrest and apoptosis.

### XR-2 and NMDi exhibit synergistic anti-tumour effects on *TP53* wild-type cancer cells

**Fig. 3:**
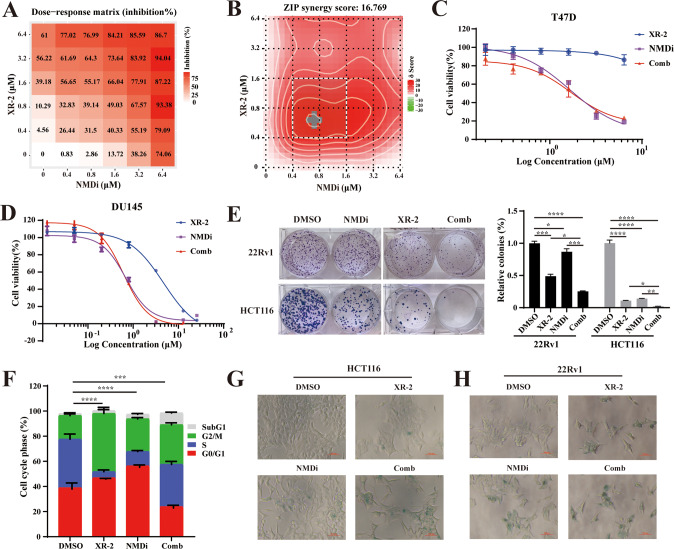
Combination of XR-2 with NMDi exhibited synergistic effects on *TP53* wild-type solid cancer cell lines. **A** Dose–response matrix of 22Rv1 cells was treated with different concentrations of NMDi and XR-2 for 72 h. **B** Zip model to evaluate the synergistic effects using data from the dose–response matrix. **C**, **D** Cell viability assay detected by CCK-8 kit of two *TP53*-mutated cell lines (**C**: T47D; **D**: DU145) treated with the indicated concentration of XR-2 and NMDi alone or in combination for 72 h. **E** Images and quantitative results of the colony-forming assay of 22Rv1 and HCT116 cells treated with NMDi or XR-2 alone or in combination. HCT116 cells were treated with 3.2 µM XR-2 and 3.2 µM NMDi alone or in combination, and 1.6 µM of these two agents were incubated with 22Rv1 cells. (Data are mean ± standard deviation, and *p*-values were calculated by the ungrouped *t*-test. *p ≤ 0.05, **p ≤ 0.01, ***p ≤ 0.001, ****p ≤ 0.0001; ns not significant. *N* = 3) **F** Analysis of the proportion of cells in each cell-cycle stage by propidium iodide staining in 22Rv1 cells treated with 3.2 µM XR-2 and 3.2 µM NMDi alone or in combination for 24 h. Statistical analysis was conducted using a chi-square test, and DMSO-treated cells were used as control. (Data are mean ± standard deviation, and *p*-values were calculated using ANOVA with Tukey HSD post hoc test. *p ≤ 0.05, **p ≤ 0.01, ***p ≤ 0.001, ****p ≤ 0.0001; ns not significant. *N* = 3) **G**, **H** Representative images of 22Rv1 and HCT116 cells treated with XR-2 and NMDi alone or in combination for 96 h followed by β-galactosidase staining. The concentration of these two agents was 1 µM for 22Rv1 and 0.2 µM for HCT116 cells. Comb: combination treatment group.

Previous studies have revealed that p53β can enhance the transactivation of p53α by forming a complex [24]. To investigate whether the apoptosis could be further increased by combined treatment of XR-2 and NMDi, a dose–response matrix was generated of 22Rv1 cells treated with different concentrations of XR-2 or NMDi alone or in combination and further calculated the magnitude of synergistic effects using Zip model (Fig. 3A, B). The results of the Zip model revealed a synergy score of 16.769, which showed a significant synergistic effect.

To determine whether the NMDi and XR-2 combination shows a synergistic effect in *TP53-*mutated cells, four *TP53*-mutated cell lines (DU145, Huh7, T47D and T24) were treated with different concentrations of NMDi and XR-2, alone or in combination. As shown in Fig. 3C, D and Fig. S2A–C, *TP53-*mutated cells treated with these two agents did not present a synergistic effect since the combined treatment did not further enhance apoptosis in cells.

Furthermore, the influence of combined treatment on the colony-forming ability of 22Rv1 and HCT116 cells was studied (Fig. 3E). Consistent with previous results, 22Rv1 or HCT116 cells treated with XR-2 or NMDi showed a significant decrease in colony-forming ability (*p* < 0.0001) compared to DMSO-treated cells. The cells treated with the combination of XR-2 and NMDi nearly did not form colonies. Further, cell-cycle analysis was performed to study the effect of these two agents on cell-cycle progression (Fig. 2F). The results suggested that XR-2 and NMDi significantly decreased the proportion of cells in the S phase and blocked cells in the G0/G1 and G2/M phases. The combination treatment with XR-2 and NMDi decreased the number of cells in the G0/1 phase, increased the number of cells in the G2/M phase and subG1 phase, and disrupted the pattern of the cell cycle (Fig. S2D). β-galactosidase staining assay was conducted to further evaluate cell senescence. 22Rv1 and HCT116 cells treated with XR-2 or NMDi for 96 h had an increase in the proportion of β-galactosidase-positive cells, and combined treatment with these two agents led to a synergistic upregulation of β-galactosidase-positive cells (Fig. 2G, H). Altogether, these data suggest the synergistic effects of combined treatment with NMDi and XR-2 on *TP53* wild-type cancer cells were due to increased cell apoptosis and senescence, and cell-cycle arrest.

### Combination treatment of XR-2 and NMDi synergistically induces cell apoptosis and activates the p53 pathway in *TP53* wild-type cancer cells

**Fig. 4:**
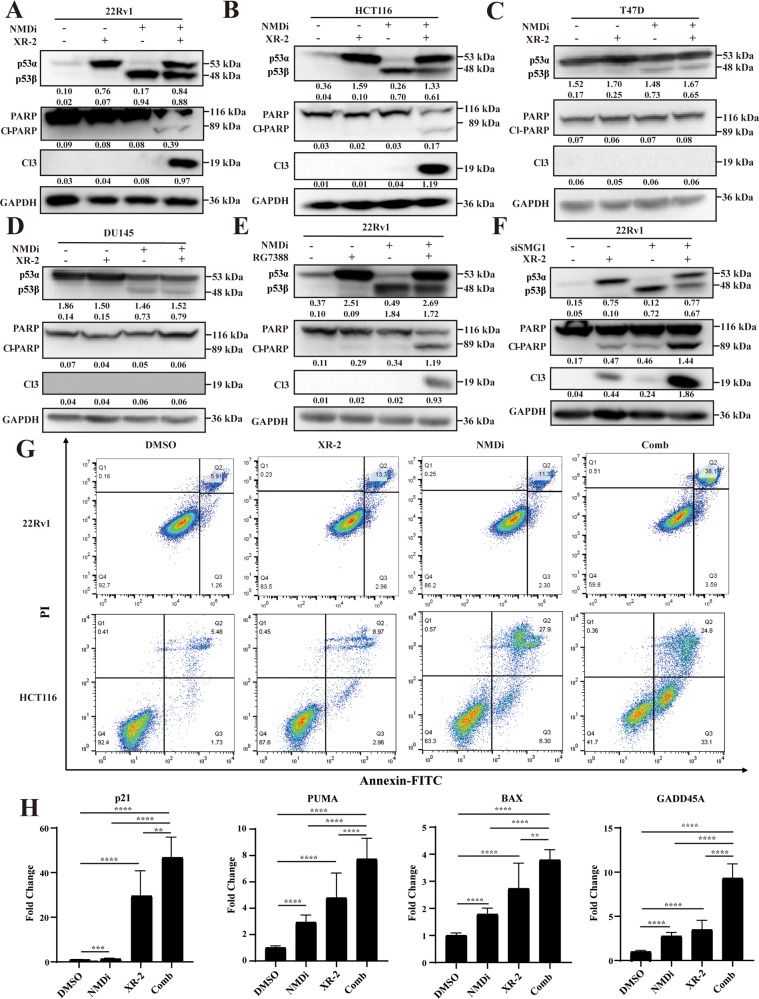
NMDi combined with XR-2 synergistically induced cell apoptosis and activated the p53 pathway in *TP53* wild-type cancer cells. **A** 22Rv1 and **B** HCT116 cells treated with 3.2 µM XR-2 and 3.2 µM NMDi alone or in combination for 24 h, and apoptosis biomarkers (cleaved PARP and cleaved Caspase 3), p53 isoforms and GAPDH protein levels were detected by Western blot analysis. **C**, **D** Two *TP53*-mutated cell lines T47D and DU145 treated with 3.2 µM XR-2 and 3.2 µM NMDi alone or in combination for 24 h, and the protein level of apoptosis markers and p53 isoforms were analyzed by Western blot. **E** 22Rv1 cells treated with siSMG1 and 3.2 µM XR-2 for 24 h, and the protein levels of apoptosis markers and p53 isoforms were measured by Western blot. **F** Protein levels of apoptosis markers and p53 isoforms of 22Rv1 cells treated with 3.2 µM NMDi and 3.2 µM RG7388 alone or in combination for 24 were measured by Western blot. **G** Flow cytometry for analyzing cell apoptosis in 22Rv1 and HCT116 cells treated with 3.2 µM XR-2 or 3.2 µM NMDi alone or in combination for 24 h using Annexin V-FITC-PI staining. **H** Fold change in mRNA expression of p53 target genes *P21*, *PUMA*, *BAX* and *GADD45A* in 22Rv1 cells treated with 3.2 µM NMDi or XR-2 alone or in combination for 24 h using qPCR. Cl-PARP cleaved PARP, Cl3 cleaved Caspase 3. Comb: combination treatment group. (Data are mean ± standard deviation, and *p*-values were calculated by the ungrouped *t*-test. *p ≤ 0.05, **p ≤ 0.01, ***p ≤ 0.001, ****p ≤ 0.0001; ns not significant. *N* = 3).

Further, Western blot was performed to study protein expression of apoptosis markers and p53 isoforms (p53α and p53β) following combination treatment. As shown in Fig. 4A, B, 22Rv1 and HCT116 cells treated with NMDi or XR-2 for 24 h slightly or barely increased the expression of cell apoptosis biomarkers. When the cells were treated with a combination of NMDi and XR-2, a dramatically increase in the expressions of cell apoptosis markers was observed. Thus, a significant synergistic apoptosis-promoting effect was mediated by combining these two agents. To confirm whether all the *TP53* wild-type cancer cells had similar synergistic effects or were the effect cell line specific, hence two additional *TP53* wild-type cancer cell lines (RT4 and MCF7) were treated with XR-2 and NMDi alone or in combination (Fig. S3A, B). The synergistic effects were also present in these two *TP53* wild-type cancer cell lines as an increase in the expressions of cell apoptosis markers. In these four *TP53* wild-type cancer cells, the combined treatment increased the expressions of both p53α and p53β.

*TP53*-mutated cell lines (DU145, Huh7, T47D and T24) expressed apoptosis markers on the treatment of XR-2 and NMDi alone or in combination were also investigated. However, combined treatment of XR-2 and NMDi did not increase apoptosis in any of these four *TP53*-mutated cell lines (Fig. 4C, D, Fig. S3C, D), further confirming that the synergistic effects of XR-2 and NMDi were specific to *TP53* wild-type cancer cell lines. To rule out the off-target effect of NMDi, the expression of *SMG1* was knockdown using siRNA. As displayed in Fig. 4E, when SMG1 knockdown 22Rv1 cells treated with XR-2, the expression of apoptotic markers were significantly increased compared to control cells, indicating a synergistic effect. *SMG1* knockdown also increased the expression level of p53β (Fig. S3E, F). Similarly, MDM2 inhibitor RG7388 was used to rule out the off-target effect of XR-2 and corroborate these results further. Combined treatment with RG7388 and NMDi also significantly increased apoptosis markers, confirming the synergistic effects of these two agents (Fig. 4F).

Furthermore, we evaluated cell apoptosis using the Annexin V-FITC-PI apoptosis detection kit. As shown in Fig. 4G, treatment of 22Rv1 cells with XR-2 or NMDi for 24 h caused apoptosis of cells by 13.3% and 13.6%, respectively, and the combination treatment of these two agents increased apoptotic cells to 39.7%. In HCT116 cells, treatment with XR-2 or NMDi showed apoptosis in 11.9% and 36.2% of cells, respectively, and the combination treatment increased apoptotic cells up to 59.6%. Notably, the combination treatment increased the number of cells in later-stage apoptosis in 22Rv1 cells, and more cells were observed in early-stage apoptosis in HCT116 cells.

Figure 1 and Fig. 2 demonstrated that XR-2 and NMDi treatment could induce the expressions of p53α and p53β, respectively, and activate p53 downstream genes to varying degrees. To test whether the combination treatment could further activate the p53 pathway, qPCR was conducted to measure the mRNA levels of key p53 target genes in 22Rv1 and HCT116 cells treated with XR-2 and NMDi alone or in combination. Figure 4H and Fig. S3G demonstrated that the combined treatment could further activate the p53 pathway since the mRNA level of *GADD45* *A*, *PUMA*, *BAX* and *P21* were significantly increased (*p* < 0.0001) on the combined treatment of XR-2 and NMDi compared to DMSO-treated and single-treated cells. This confirmed that NMD inhibitors combined with MDM2 inhibitors presented synergistic effects in *TP53* wild-type cancer cells, significantly increasing cell apoptosis and activating the p53 pathway.

### Synergistic effects of XR-2 and NMD inhibitors on the cooperation of p53α and p53β

**Fig. 5:**
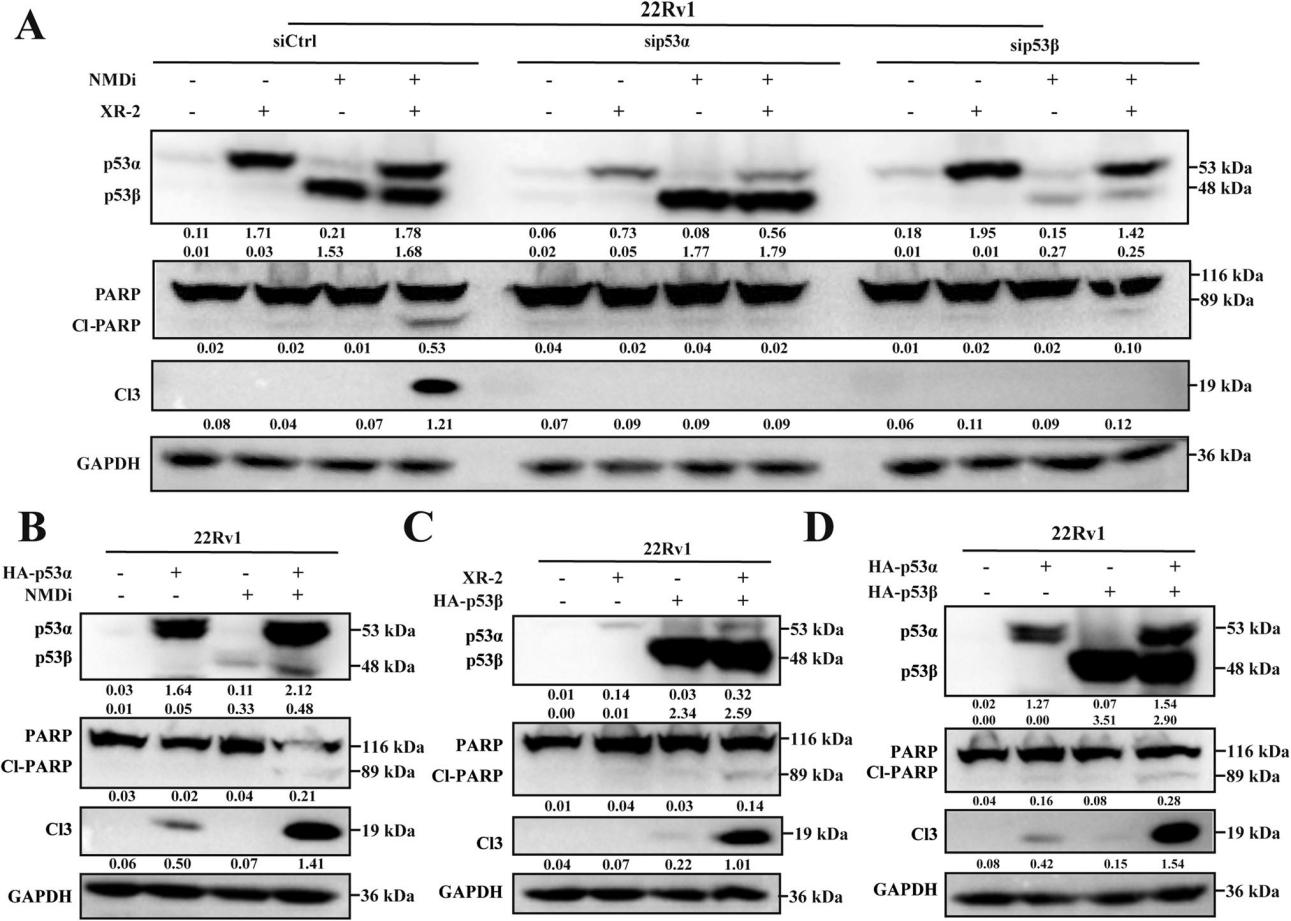
Synergistic effect of the combination of XR-2 and NMDi is dependent on the cooperation between p53α and p53β. **A** 22Rv1 cells were treated with siCtrl, sip53α, sip53β and the cells were treated with 3.2 µM NMDi and 3.2 µM XR-2 alone or in combination for 24 h. Western blot was used to detect the protein levels of apoptosis markers and p53 isoforms. **B** 22Rv1 cells were transfected with 0.3 µg PCDH–HA–p53α or PCDH backbone plasmid, followed by DMSO or 3.2 µM NMDi incubation for 24 h. The protein levels of apoptosis markers and p53 isoforms were analyzed by Western blot. **C** 22Rv1 cells were transfected with 0.3 µg PCDH–HA–p53β or PCDH backbone plasmid. The cells were incubated with DMSO or 3.2 µM XR-2 treatment for 24 h, and the protein level of apoptosis markers and p53 isoforms were measured by Western blot. **D** Overexpression of p53α or p53β alone or in combination by 0.3 µg plasmid transfection on 22Rv1 cells. Western blot analysis was used to detect the protein level of apoptosis markers and p53 isoforms. Comb: combination treatment group. Cl-PARP cleaved PARP, Cl3 cleaved Caspase 3.

XR-2 activates p53α and is depends on the *TP53* status, whereas NMDi activates p53β and is partially depends on p53β. In order to check if the mechanism of synergistic effects relies on the interaction of p53α and p53β, their expression was knockdown using siRNA in 22Rv1 cells. The knockdown and siRNA control cells were then treated with XR-2 and NMDi alone or in combination. As shown in Fig. 5A, the expression levels of apoptosis markers were increased when treated siControl cells with XR-2 and NMDi, confirming the synergistic effect. However, the synergistic effects were partially rescued by the knockdown of p53α or p53β, with a decrease in the expression of apoptosis markers (Fig. 5A). These results demonstrated that the synergistic effects depended on the intact cooperation between p53α and p53β, and any changes in either p53 isoforms could negate the synergistic effects.

Subsequently, two plasmids (PCDH–HA–p53α and PCDH–HA–p53β) were constructed. 22Rv1 and HCT116 cells were transfected with PCDH–HA–p53α to mimic the XR-2 effect, followed by NMDi treatment. As demonstrated in Fig. 5B and Fig. S4A, the combination of NMDi treatment and p53α overexpression increased the expression of apoptosis markers, thereby demonstrating a synergistic effect. The combination of p53β overexpression and XR-2 treatment in 22Rv1 and HCT116 cells also increased the expression of apoptosis markers, which implied a significant synergistic effect of the combination treatment (Fig. 5C, Fig. S4B). Finally, 22Rv1 and HCT116 cells were overexpressed with p53α and p53β alone or in combination. A synergistic effect was also observed in the cells on the co-expression of p53α and p53β (Fig. 5D, Fig. S4C). These results show that the synergistic effect of the combination treatment of XR-2 and NMDi relies on the cooperation of p53α and p53β, specific to *TP53* wild-type cancer cell lines.

### RNA-sequencing reveales molecular mechanisms underlying the synergistic effects

**Fig. 6:**
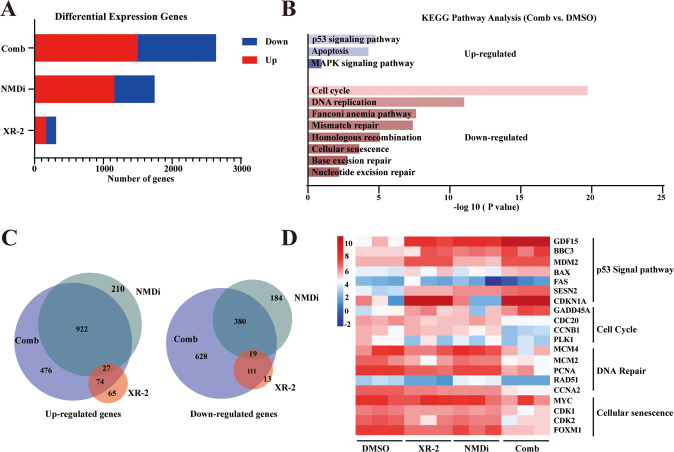
RNA-sequencing analysis of 22Rv1 cells treated with NMDi or XR-2 alone or in combination. 22Rv1 cells were treated with 3.2 µM XR-2 and 3.2 µM NMDi alone or in combination for 24 h in triplicate. **A** Numbers of DEGs in the single or combination treatment groups. All DEGs were compared between the drug-treated groups and DMSO-treated groups. **B** KEGG analysis results of the most significantly enriched pathways in the combination treatment group. **C** Venn diagram of upregulated and downregulated genes in the single or combination treatment groups. **D** The heatmap exhibited the representative genes of the p53 signal pathway, DNA repair pathway, cell-cycle pathway and cellular senescence pathway in single or combination treatment groups. Comb: combination treatment group. *N* = 3.

To identify the molecular mechanisms underlying the synergistic effects of the combination treatment with XR-2 and NMDi, transcriptional profile short-read sequencing of 22Rv1 cells was carried out after 24 hours of treatment with DMSO, XR-2 and NMDi alone or in combination. Compared to the single treatment, combination treatment led to more DEGs, which implied additional changes in the transcriptional profile (Fig. 6A). XR-2 treatment showed activation of the p53 signal pathway, and the enrichment of DNA replication, cell cycle, cellular senescence and DNA damage repair pathway, including base excision repair, mismatch repair and nucleotide excision repair (Fig. S4A). NMDi treatment markedly enriched the apoptosis pathway and p53 signalling pathway, and also enriched genes related to two important signalling pathways in cancers, cAMP and Rap1 (Fig. S4B). The combination treatment enriched nearly all the pathways activated by the single treatment group. Furthermore, pathways that were not enriched in the single treatment group had extensive crosstalk with the p53 signalling pathway (i.e. mitogen-activated protein kinase) (Fig. 6B). Further, the Venn analysis was performed to compare the changes in their DEGs (Fig. 6C). The DEGs of the combination treatment groups covered most of the genes enriched in the single treatment groups. However, the expression of unique genes was observed. The KEGG analysis demonstrated that the downregulated unique genes were similar to those in the XR-2-treated groups (Fig. S4C). These unique upregulated genes were related to metabolism, such as the metabolism of alpha-linolenic acid and choline in cancer (Fig. S4D). Subsequently, a heatmap was generated to show the expression level of some important genes. As shown in Fig. 6D, the combined treatment further enhanced the expression of the p53 signalling pathway genes, including *GDF15*, *BBC3* (*PUMA* coding gene), *CDKN1A* (*P21* coding gene) and *BAX* and downregulated genes that were involved in cell-cycle progressions, such as *CDC20* and *CCNB1*. Genes related to DNA damage repair and cellular senescence were downregulated on a combination treatment of these two agents as well.

## Discussion

**Fig. 7:**
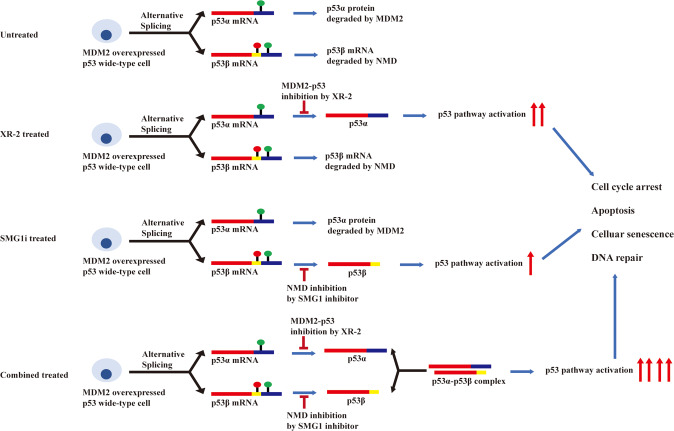
The mechanism of nonsense-mediated mRNA decay inhibition synergizes with MDM2 inhibition to suppress *TP53* wild-type cancer cells.

The p53 dysfunction plays an essential role in tumorigenesis and tumour development. Hence, reactivating and restoring the p53 function can serve as a powerful and promising strategy in cancer therapeutics. This study used a combination of NMDi and XR-2 in MDM2-overexpressed *TP53* wild-type cancer cells and synergistically promoted cell apoptosis and DNA repair, inhibited cell-cycle progression and suppressed cell proliferation (Fig. 7).

As a potential target in cancer therapeutics, NMD inhibition can stimulate tumour immune response [26, 27], decrease genome instability [25], and activate the p53 pathway [11]. It is reported that compound 11j (a SMG1 inhibitor) could increase the expression of p53β and p53γ and reactivate the p53 pathway in MDM2-overexpressed or *TP53* mutations downstream of exon 9 in tumour cells [11]. Results of our study also revealed that NMDi mainly increased the expressions of p53β and transactivated p53 target genes. The KEGG analysis revealed that the p53 pathway was significantly enriched in the NMDi-treated group. These results emphasized the function of p53β and the potential use of NMDi in cancer treatment via the p53 pathway.

MDM2 inhibitors have been reported to combine with other agents for cancer therapies, such as CDK4/6 inhibitors [28] and 5-azacitidine [29]. The p53β could enhance p53α transactivation ability by direct or indirect means [24, 30]. Hence, this study showed that simultaneous activation of p53α and p53β by agents or overexpression had a significant synergistic effect. Therefore, it is tempting to believe that the mechanism underlying the synergistic effect is partly based on the cooperation of p53α and p53β. This study also provided rationales for the designation of MDM2 inhibitors combination therapy. However, the specific interaction pattern of p53α and p53β need to be further studied.

Our results demonstrated that the synergistic effect of the NMDi and MDM2 inhibitor was only observed in *TP53* wild-type cancer cells and not in *TP53-*mutated cells. NMD inhibition or read-through agents were used to increase the expression of some proteins with nonsense mutations [31–33]. A study combined a read-through drug and an MDM2 inhibitor to restore the function of nonsense mutant *TP53*, and synergistic effects were observed [31]. NMD could degrade *TP53* mutants with nonsense mutation downstream of exon 9 according to 50–55nt rules [11], thus the strategy of combining NMD and MDM2 inhibition could achieve synergistic effects in the subgroup of cancers with *TP53* mutation. The restored nearly full-length p53 protein could coordinate with p53β, thus further activating the p53 pathway. However, *TP53* mutation sites of cell lines in this study were located in the prominent mutation domain, DNA-binding domain [34], and no synergistic effects of the combination treatment of NMDi and XR-2 were observed. Nevertheless, it would still be meaningful to try this combination strategy in these *TP53-*mutated cancers.

However, more studies in animal models are required to explore the effects and safety of NMD inhibitors combined with MDM2 inhibitors before they are tested in humans. Nevertheless, this finding offers insight into the future design of drug combinations of NMD and MDM2 inhibitors in cancer treatment.

## Materials and methods

### Cell culture and reagents

Human tumour cell lines 22Rv1, RT4, DU145 and T24 were cultured in Roswell Park Memorial Institute (RPMI) 1640 medium (Meilunbio, Dalian, China) supplemented with 10% fetal bovine serum (FBS) (Thermo Fisher Scientific, MA, USA) and a cocktail of antibiotics (Beyotime Biotechnology, Shanghai, China). MCF7, T47D, Huh7 and HCT116 were cultured in Dulbecco’s Modified Eagle Medium (DMEM) (Meilunbio, Dalian, China) containing 10% FBS, 100 U/mL penicillin and 100 mg/mL streptomycin. All cells were purchased from ATCC and cultured in a humidified incubator at 37 °C with 5% CO2.

The MDM2 inhibitor XR-2 was designed and synthesized by Wu et al. [22]. The NMD inhibitor was synthesized according to the structure of the previous study [25]. RG7388 was purchased from Selleck (Selleck Chemicals, TX, USA). All compounds were dissolved in dimethyl sulfoxide (DMSO) and stored at −20 °C until further use.

### Western blot

Cells grown in six-well plates were washed once with cold phosphate-buffered saline (PBS), and lysis buffer (0.05 M Tris (pH 6.8), 2% sodium dodecyl sulphate, 10% glycerol, 0.1% bromophenol blue and 5% β-mercaptoethanol) was directly added to the cell monolayer. Cell lysates were collected, boiled at 95 °C for 15 min and centrifuged at 12,000 × *g* for 5 min. Proteins from the supernatant of the lysate were loaded on 10% or 12% sodium dodecyl sulphate–polyacrylamide electrophoresis gel (SDS-PAGE) and then electro-transferred to 0.45 µM polyvinylidene fluoride (PVDF) membranes. Membranes were blocked using 0.1% casein buffer at room temperature for 1 h, followed by primary antibody incubation at 4 °C overnight. The membrane was washed thrice with tris-buffered saline with Tween 20 (TBST), followed by horseradish peroxidase-conjugated secondary antibodies incubation and finally detected with a Western Lightning chemiluminescent substrate using an imaging ChampChemi Gel Image Analysis System (Beijing, China). All the experiments were performed in triplicates. Glyceraldehyde 3-phosphate dehydrogenase (GAPDH) or actin was used as the loading control. The bands were quantified using ImageJ software (National Institutes of Health, MD, USA). Information regarding primary antibodies is listed in Supplementary Table 1.

### RNA extraction, cDNA synthesis and quantitative real-time polymerase chain reaction (qPCR)

One millilitre of TRIzol (Thermo Fisher Scientific, MA, USA) was added directly to the cell monolayer in six-well plates. Chloroform was added to separate the three layers, and the aqueous phase containing the RNA was collected, precipitated using isopropanol, washed with 70% ethanol and resuspended using the RNase-free water. NanoDrop 2000C (Thermo Fisher Scientific, MA, USA) was used to quantify RNA. The quality of RNA was evaluated using 1.2% agarose gel electrophoresis. Total RNA was reverse transcribed using a reverse-PCR kit (AGbio, Changsha, China). qPCR was performed on cDNA using 2× SYBR Green Mix (Applied Biosystems, MA, USA) according to the manufacturer’s protocol. The 2^−ΔΔCt^ method was used to calculate relative fold changes, and GAPDH was used as an endogenous control. Fold-change expressions were normalized to the expression of the DMSO-treated group. All primer sequences are listed in Supplementary Table 2.

### Cell viability assay

All cells were seeded at a density of 2000–3000 cells per well in a 96-well plate and treated with the indicated concentrations of XR-2 or NMDi for 24 h. Cell viability was measured by Cell Counting Kit-8 (CCK-8) (Meilunbio, Dalian, China) according to the manufacturer’s instructions. Luminescence was measured by the Gen5 Microplate Reader (BioTek Instruments, VT, USA). For the drug combination matrix cell viability assay, an online database SynergyFinder (version 2.0, https://synergyfinder.fimm.fi/synergy/) with the Zip model was used. The interaction between the two agents is likely to be antagonistic if the synergy score is below −10, additive if the synergy score is between −10 and 10 and synergistic if the synergy score is >10. The half-maximal inhibitory concentration (IC_50_) and dose–response curves were generated using GraphPad Prism 8 (GraphPad Software, CA, USA).

### Clonogenic assay

Clonogenic assay was used to determine cell survival after treatment with NMDi or XR-2 alone or in combination. 22Rv1 or HCT116 cells were plated in a six-well plate at a density of 800 or 1500 cells per well, respectively. Cells were treated with chemicals at the indicated concentration 24 h after plating. The media of the cells was replaced with fresh media 24 h post-treatment, and the cells were cultured for 2 weeks. The colonies were fixed with methanol, stained with 0.2% crystal violet (Meilunbio, Dalian, China) and washed with water; subsequently, the colony images were captured.

### Cell apoptosis assay

Cell apoptosis assay was performed using Annexin V/fluorescein isothiocyanate /propidium iodide (Annexin V-FITC-PI) Apoptosis Detection Kit (Meilunbio, Dalian, China). 22Rv1 and HCT116 cells seeded in a six-well plates were treated with NMDi or XR-2 alone or in combination for 24 h. Cells were trypsinized, washed twice with cold PBS and resuspended in an adequate volume of binding buffer. The cell concentration was adjusted to ~1 × 10^6^/mL. Subsequently, 100 µL of cells were taken and incubated with 5 µL of Annexin V-FITC and 5 µL of PI for 15 min at room temperature. Cells were sorted on a BD Flow Cytometer (BD Biosciences, NJ, USA) 1 h after staining. Data were processed using the FlowJo software (FlowJo LLC, OR, USA).

### Cell-cycle analysis

Cells were treated with the indicated concentration of NMDi or XR-2 alone or in combination for 24 h. Further, the cells were trypsinized and washed twice with cold PBS. The cells were fixed with 70% ethanol overnight, washed twice with cold PBS, digested using 50 µg/µL RNaseA (Beyotime Biotechnology, Shanghai, China) at 37 °C for 1 h and stained with 50 µg/µL PI at 4 °C for 30 min. Cells were sorted using a BD Flow Cytometer (BD Biosciences, NJ, USA) 1 h after staining.

### Plasmid construction and transfection

The coding sequences of the human p53α and p53β isoforms were obtained from the NCBI Gene database (p53α, NM_000546.6; p53β, NM_001126114.3). A hemagglutinin (HA) tag was added to the 5’ end of the sequence of *TP53* isoforms sequence, and the whole sequence was inserted into the PCDH backbone at XhoI and NdeI restriction endonuclease sites. All reconstructed plasmids were sequenced using Sanger sequencing. The plasmids were transfected in 22Rv1 and HCT116 cells with Lipofectamine™ 3000 Reagent (Thermo Fisher Scientific, MA, USA) according to the manufacturer’s instructions. The indicated concentrations of XR-2,NMDi, or 0.1% DMSO were added to transfected cells 6 h post-transfection. The cells were further incubated for another 24 h for protein extraction.

### siRNA and transfection

Small interfering RNA (siRNA) target for p53α, p53β, SMG1 and non-target control was designed and synthesized by Sangon Biotech (Shanghai, China). siRNA stocks (20 µM) were diluted in opti-MEM, mixed with opti-MEM diluted Lipofectamine™ RNAiMAX Transfection Reagent (Thermo Fisher Scientific, MA, USA) and incubated for 20 min at room temperature. This mixture was added to cells at a final concentration of 20–40 nM of the siRNA. The indicated concentrations of XR-2 or NMDi were added to cells 12 h post-transfection, and incubated for another 24 h followed by RNA or protein extraction.

### Cell senescence analysis

Cell senescence was evaluated based on β-galactosidase activity using a β-galactosidase staining kit (Beyotime Biotechnology, Shanghai, China). Briefly, cells were treated with XR-2 and NMDi alone or in combination for 96 h. The cells were later fixed using fixation buffer, washed with PBS and stained with β-galactosidase buffer at 37 °C overnight. β-galactosidase-positive cells were stained blue. Images were taken under 20× magnification.

### Short-read RNA-sequencing and data processing

The mRNA extracted from 2 μg of total RNA was enriched by polyT-conjugated magical beads and was then used for library preparation. The quality of the sequences was checked using the FastQC (https://github.com/s-andrews/FastQC). The raw reads that passed the quality control were aligned and assembled using STAR (https://github.com/alexdobin/STAR). Differentially expressed genes (DEGs) were analyzed using HTseq-count (https://pypi.org/project/HTSeq) and edgeR (https://bioconductor.org/packages/edgeR). Gene Ontology (GO) and Kyoto Encyclopedia of Genes and Genomes (KEGG) analyses were performed using EnrichR database (https://cran.rproject.org/web/packages/enrichR/index.html). Heat maps were generated on the HiPlot tool (https://hiplot.com.cn/basic/heatmap).

### Statistical analyses

Statistical analyses were performed in GraphPad Prism software (version 8.0.2). Comparisons between two groups were analyzed with a two-tailed, ungrouped Student’s *t*-test, except where specified. *p* ≤ 0.05 was accepted as statistically significant.

## Supplementary information

Supplementary Figure Legends

Figure S1

Figure S2

Figure S3

Figure S4

Supplementary tables

Original Data File

Original Data File

## Data Availability

The datasets of RNA-sequencing in this study are available from GSA with the accession number HRA001447.
