# Supplementary material for: Nonsense-mediated mRNA decay inhibition synergizes with MDM2 inhibition to suppress TP53 wild-type cancer cells in p53 isoform-dependent manner
Source: Cell Death Discov. 2022 Sep 30;8:402. doi: 10.1038/s41420-022-01190-3 (PMC9525646; doi:10.1038/s41420-022-01190-3)
Supplement: Supplementary file 1 — Supplementary Figure Legends [file 41420_2022_1190_MOESM1_ESM.doc]

**Fig. S1** **Combination of XR-2 with NMDi in Huh7, T24 and 22Rv1 cell lines. A** Scheme of *TP53* mutation site of four *TP53*-mutated cells. **B,C** Cell viability assay of Huh7 and T24 cells treated with indicated concentrations of XR-2 or NMDi alone or in combination for 72 hours. **D** Cell cycle patterns of 22Rv1 cells treated with XR-2 or NMDi alone or in combination using flow cytometry analysis.

**Fig. S2** **Combination of XR-2 with NMDi exhibited a synergistic effect on *TP53* wild-type cancer cell lines.** **A–D** Western blot detected that the synergistic effect of the combination treatment of 3.2 µM NMDi and 3.2 µM XR-2 for 24 hours exists in two *TP53* wild-type cancer cell lines (**A** RT4 and **B** MCF7), while it did not exist in *TP*53-mutated cell lines (**C** Huh7 and **D** T24). **E,F** mRNA level of p53β and SMG1 using siSMG1. **G** mRNA expression of key p53 target genes *P21*, *PUMA*, *BAX* and *GADD45A* in HCT116 cells treated with 3.2 µM NMDi and 3.2 µM XR-2 alone or in combination for 24 hours. **(**Data are Mean ± standard deviation, and p-values were calculated by ungrouped t-test. *p ≤ 0.05, **p ≤ 0.01, ***p ≤ 0.001, ****p ≤ 0.0001; ns, not significant. N=3)

**Fig. S3 KEGG analysis of RNA sequencing analysis of 22Rv1 cells. A** KEGG analysis of XR-2 treated groups. **B** KEGG analysis of NMDi-treated groups. **C** KEGG analysis of the unique up-regulated genes in the combination groups. **D** KEGG analysis of the unique down-regulated genes in the combination groups. KEGG, Kyoto Encyclopedia of Genes and Genomes.

**Fig. S4 Synergistic effect of the combination of XR-2 and NMDi is dependent on the cooperation between p53α and p53β in HCT116 cells**. **A** Western blot results of the combination of p53α overexpression and 3.2 µM NMDi treatment for 24 h showed a synergistic effect on HCT116 cells. **B** The combination of p53β overexpression and 3.2 µM XR-2 treatment for 24 h on HCT116 cells. **C** Co-overexpression of p53α and p53β showed a synergistic effect on HCT116 cells. Cl-PARP, cleaved PARP; Cl3, cleaved Caspase 3.
