# Supplementary figures and images for: Nonsense-mediated mRNA decay inhibition synergizes with MDM2 inhibition to suppress TP53 wild-type cancer cells in p53 isoform-dependent manner
Source: Cell Death Discov. 2022 Sep 30;8:402. doi: 10.1038/s41420-022-01190-3 (PMC9525646; doi:10.1038/s41420-022-01190-3)

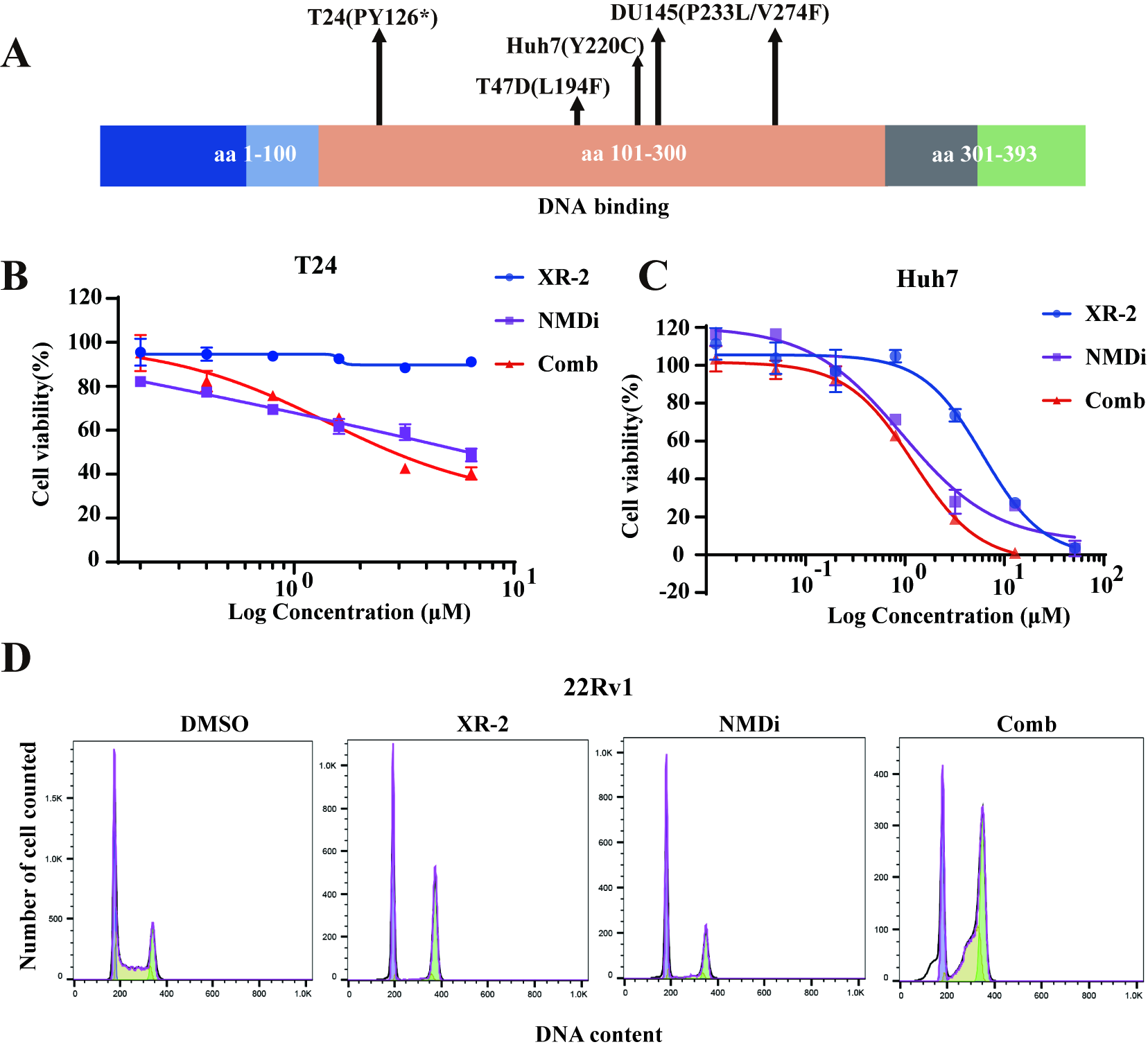

Supplement: Supplementary file 2 — Figure S1 [file 41420_2022_1190_MOESM2_ESM.tif]

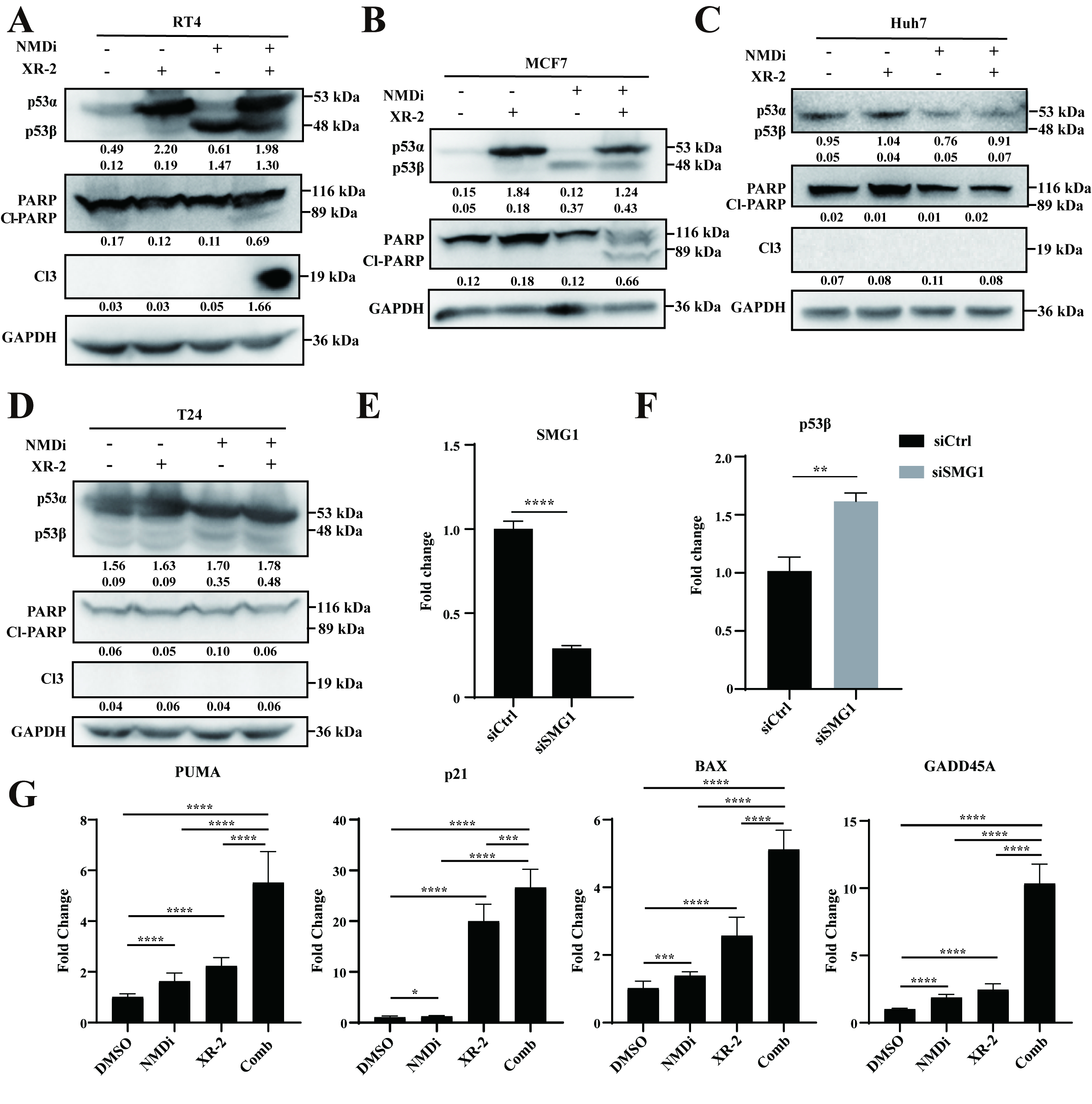

Supplement: Supplementary file 3 — Figure S2 [file 41420_2022_1190_MOESM3_ESM.tif]

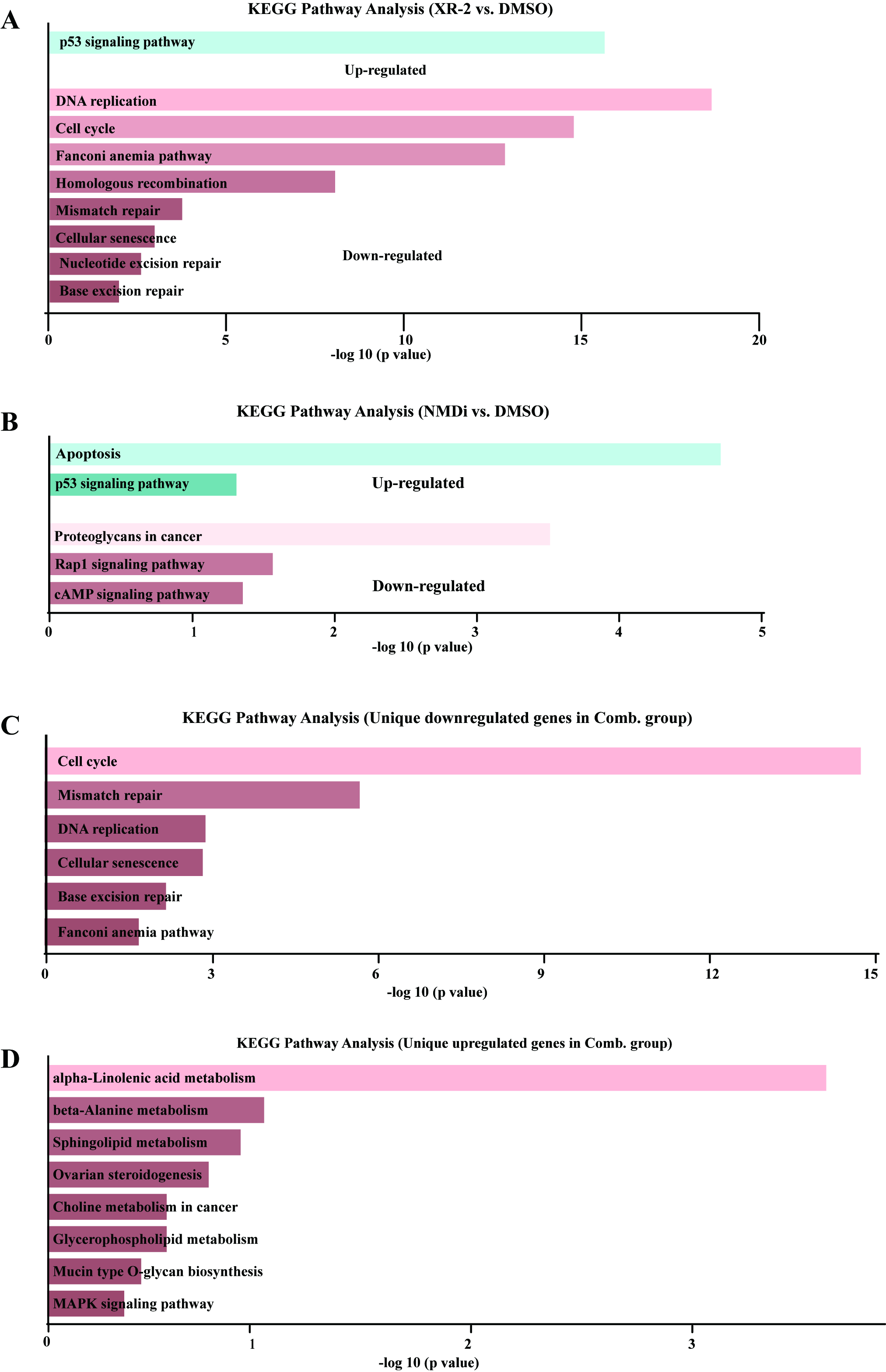

Supplement: Supplementary file 4 — Figure S3 [file 41420_2022_1190_MOESM4_ESM.tif]

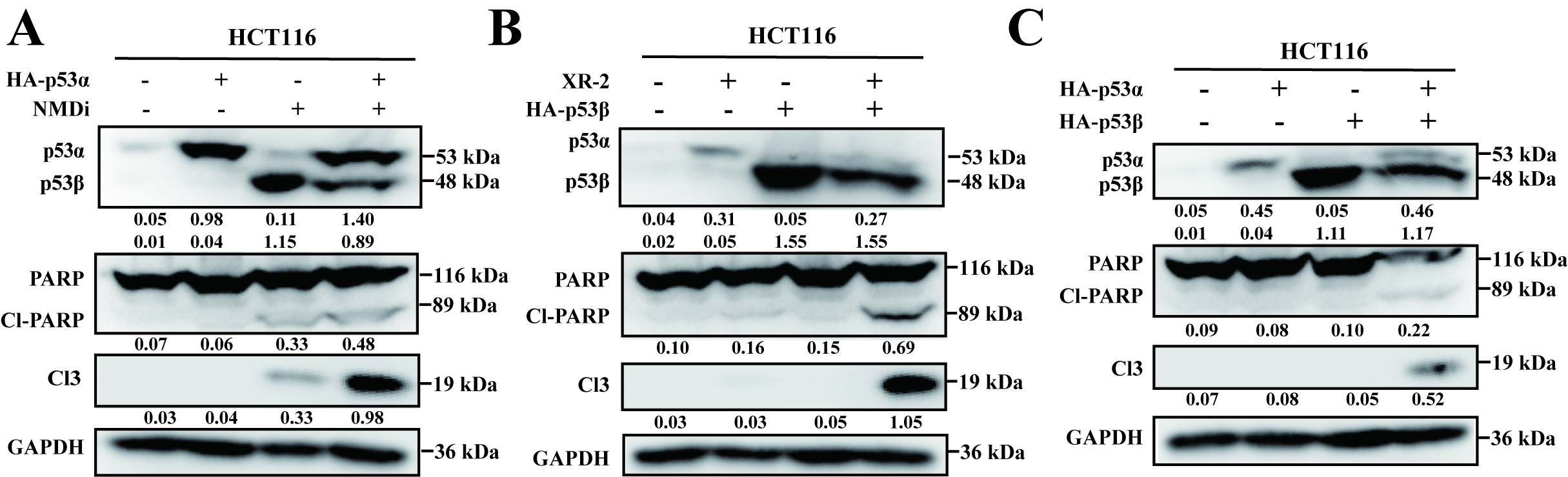

Supplement: Supplementary file 5 — Figure S4 [file 41420_2022_1190_MOESM5_ESM.tif]
