# Supplementary material for: Nonsense-mediated mRNA decay inhibition synergizes with MDM2 inhibition to suppress TP53 wild-type cancer cells in p53 isoform-dependent manner
Source: Cell Death Discov. 2022 Sep 30;8:402. doi: 10.1038/s41420-022-01190-3 (PMC9525646; doi:10.1038/s41420-022-01190-3)
Supplement: Supplementary file 6 — Supplementary tables [file 41420_2022_1190_MOESM6_ESM.docx]

Supplementary table 1: primary antibodies

|  | Company | lot. |
| --- | --- | --- |
| β-actin | Abmart | M20011 |
| p53 | Abcam | Ab26 |
| Cleaved Caspase-3 | CST | 9664 |
| PARP and cleaved PARP | Santa Crui | sc-365315 |
| HA-tag | Abmart | M20003 |

Supplementary table 2: primer sequence of real-time PCR

| Transcripts and primer orientation | Sequence |
| --- | --- |
| p53-F | CAGGGAGCACTAAGCGA |
| p53α-R | CCACGGATCTGAAGGGT |
| p53β-R | CAGGCAAAGTCATAGAACCA |
| p53γ-R | GTCAAGTAGCATCTGAAGGGTG |
| PUMA-F | GACGACCTCAACGCACAGTA |
| PUMA-R | AGGAGTCCCATGATGAGATTGT |
| GADD45A-F | CTGGAGGAAGTGCTCAGCAAAG |
| GADD45A-R | AGAGCCACATCTCTGTCGTCGT |
| BAX-F | TCAGGATGCGTCCACCAAGAAG |
| BAX-R | TCAGGATGCGTCCACCAAGAAG |
| p21-F | ATGAAATTCACCCCCTTTCC |
| p21-R | CCCTAGGCTGTGCTCACTTC |
| SMG1-F | CTGGCAACCCAGAACTGATAG |
| SMG1-R | CTGGCAACCCAGAACTGATAG |

Supplementary table 3: sequence of siRNA

| Name of siRNA | sequence |
| --- | --- |
| sip53-sense | GCAUCUUAUCCGAGUGGAATT |
| sip53-antisense | UUCCACUCGGAUAAGAUGCTT |
| sip53β-sense | GGACCAGACCAGCUUUCAATT |
| sip53β-antisense | UUGAAAGCUGGUCUGGUCCTT |
| SMG1-sense | GCCGAGAUGUUGAUCCGAAUATT |
| SMG1-antisense | UAUUCGGAUCAACAUCUCGGCTT |
