# Supplementary material for: Nonsense-mediated mRNA decay inhibition synergizes with MDM2 inhibition to suppress TP53 wild-type cancer cells in p53 isoform-dependent manner
Source: Cell Death Discov. 2022 Sep 30;8:402. doi: 10.1038/s41420-022-01190-3 (PMC9525646; doi:10.1038/s41420-022-01190-3)

Figure 3G

HCT116-comb

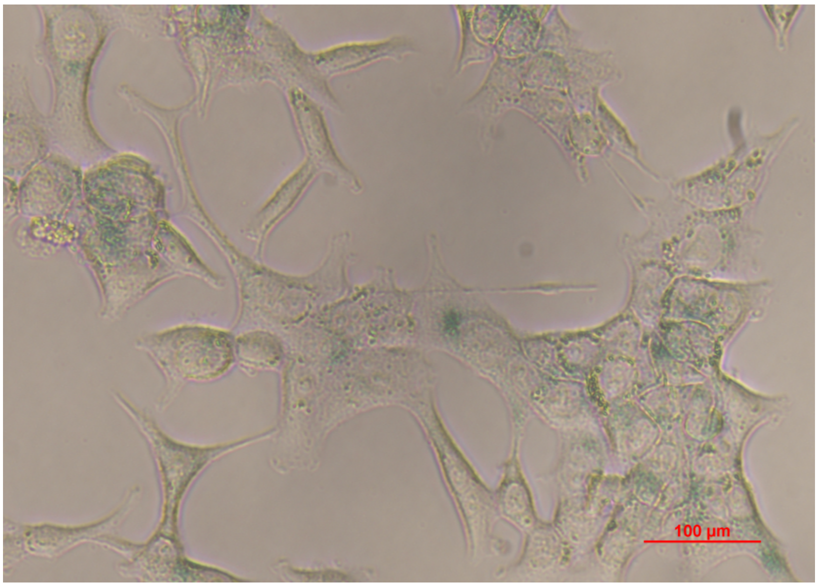

HCT116-NMDi

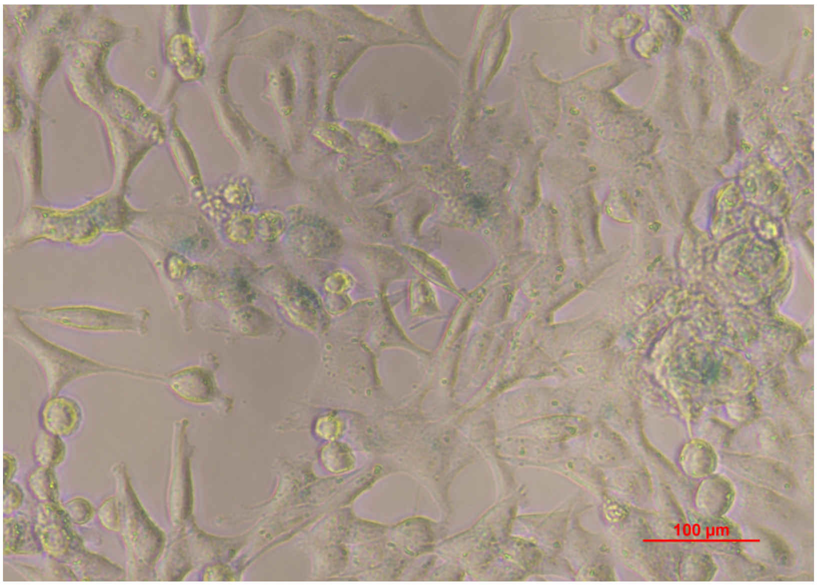

HCT116-XR-2

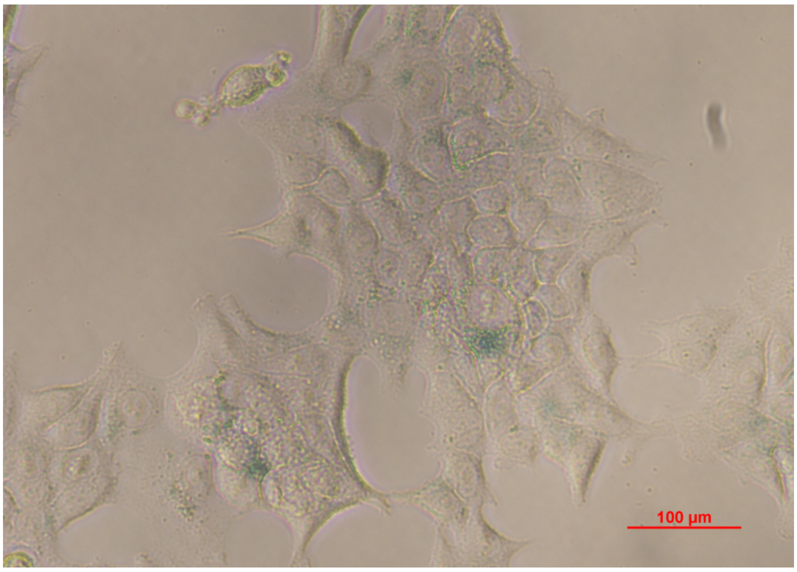

HCT116-DMSO

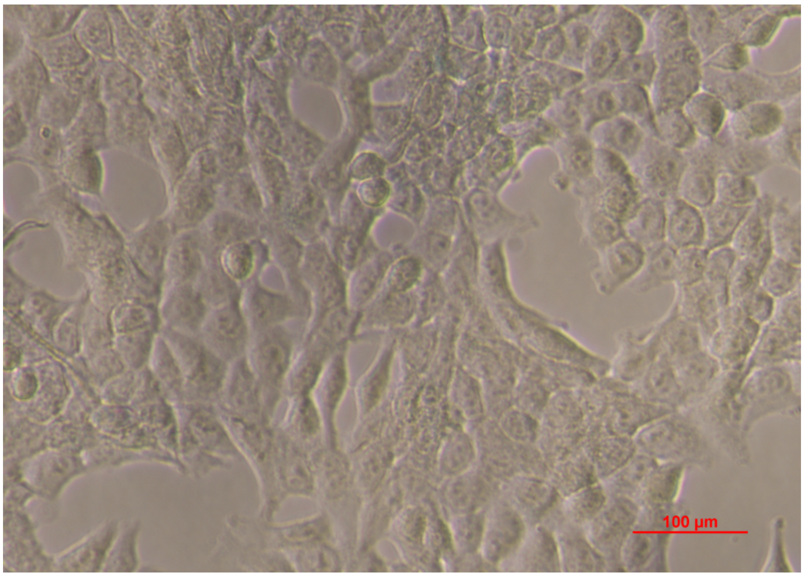

Figure 3H

22Rv1-DMSO

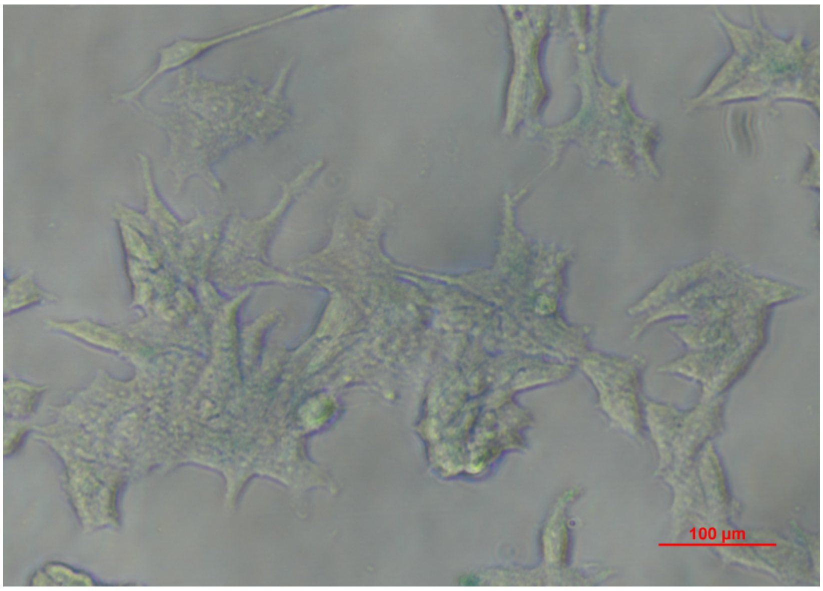

22Rv1-XR-2

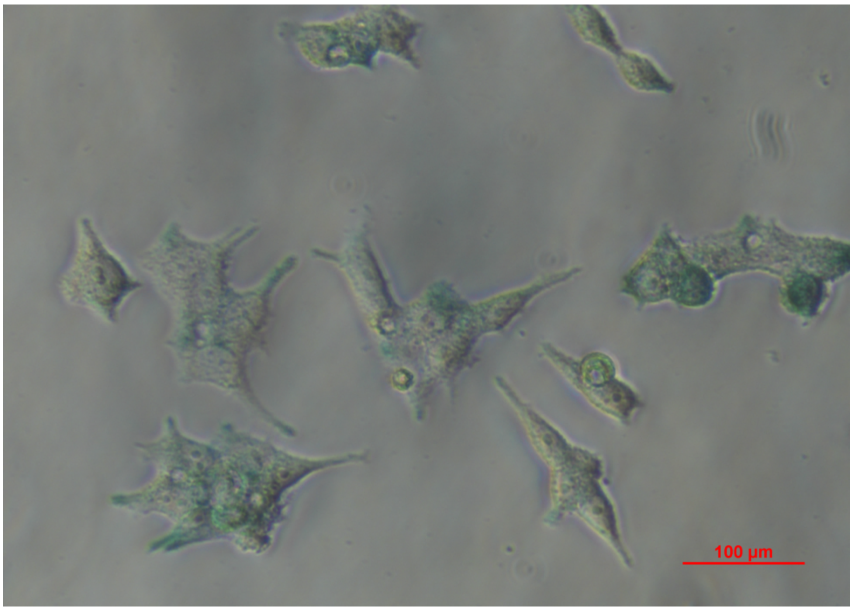

22Rv1-NMDi

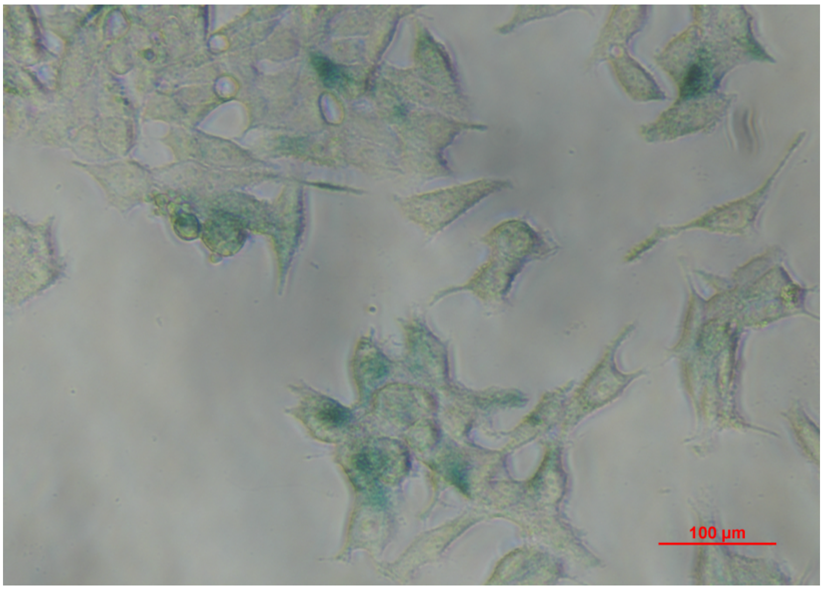

22Rv1-Comb

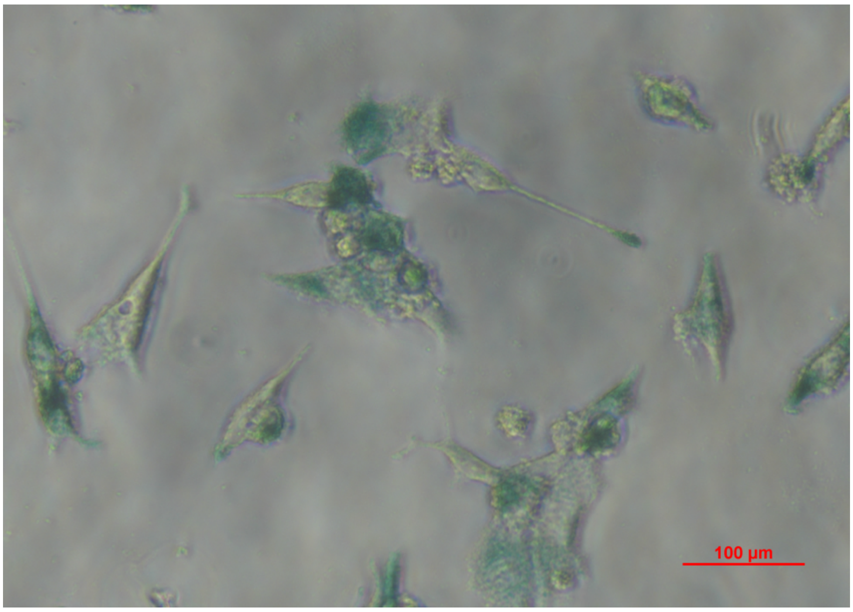

Raw data-colony forming

Figure 1C 22Rv1

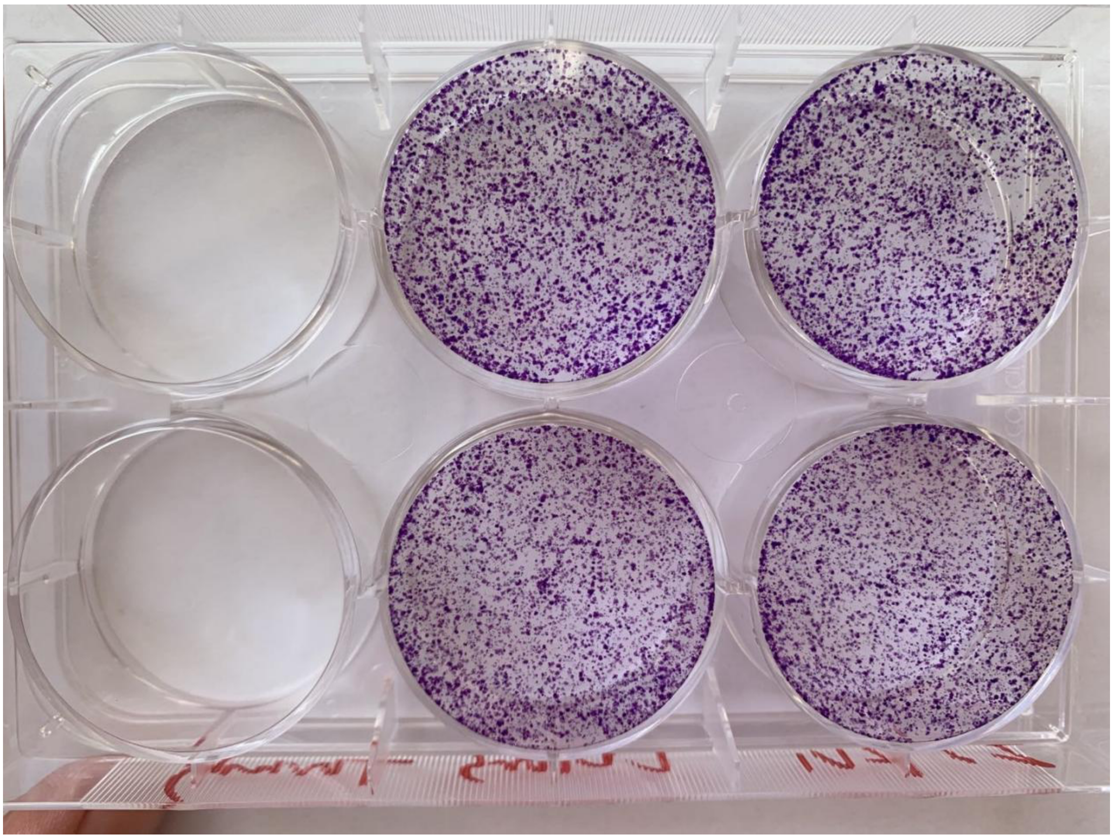

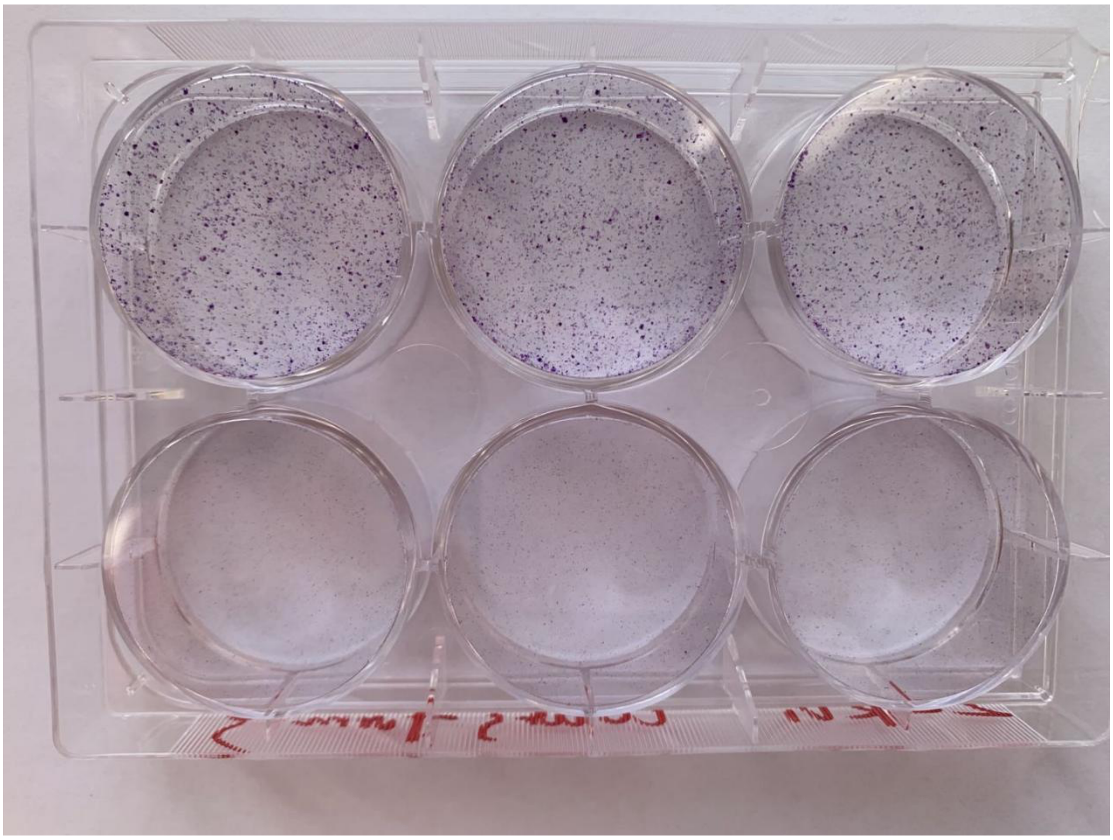

Figure 1C HCT116

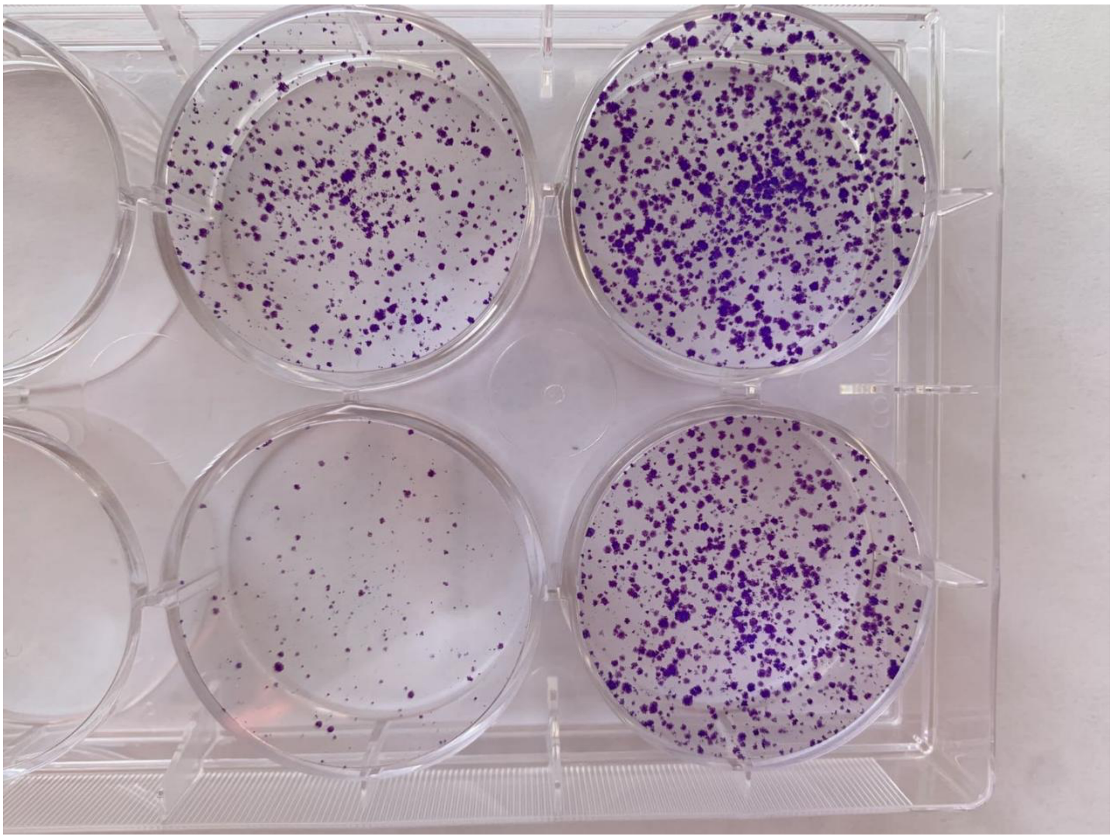

Figure 3E

22Rv1

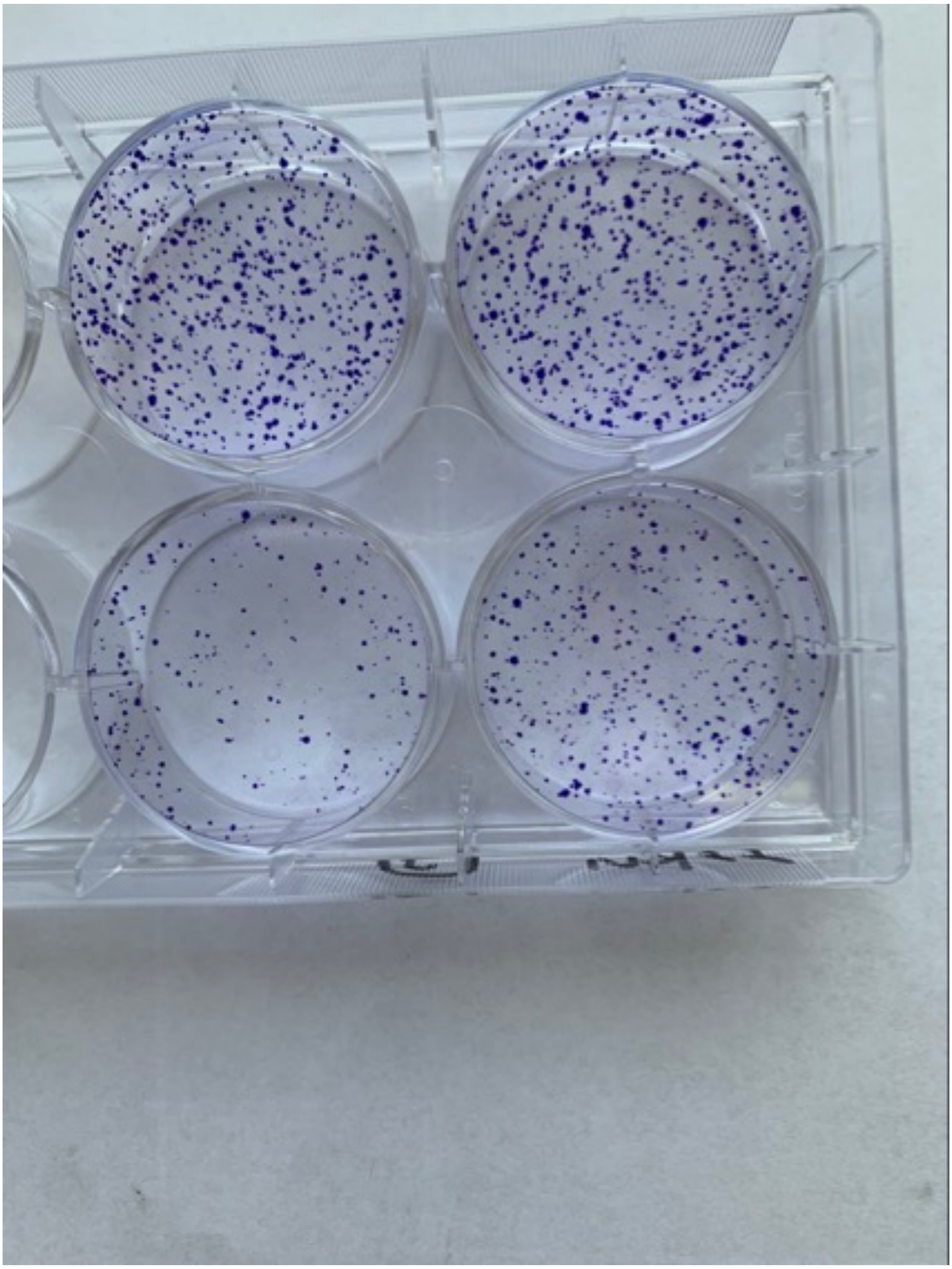

HCT116

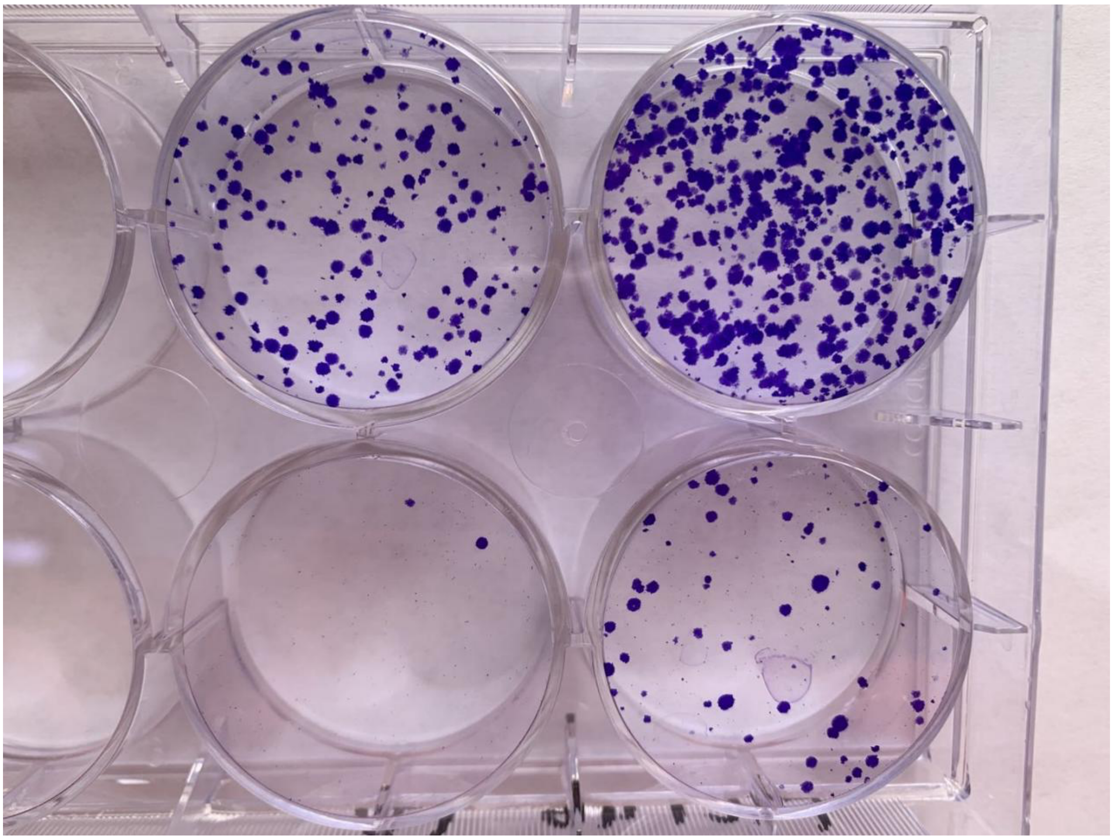

Supplement: Supplementary file 7 — Original Data File [file 41420_2022_1190_MOESM7_ESM.pdf]
