# Supplementary material for: Nonsense-mediated mRNA decay inhibition synergizes with MDM2 inhibition to suppress TP53 wild-type cancer cells in p53 isoform-dependent manner
Source: Cell Death Discov. 2022 Sep 30;8:402. doi: 10.1038/s41420-022-01190-3 (PMC9525646; doi:10.1038/s41420-022-01190-3)

original gel and blot

Figure 1E GAPDH

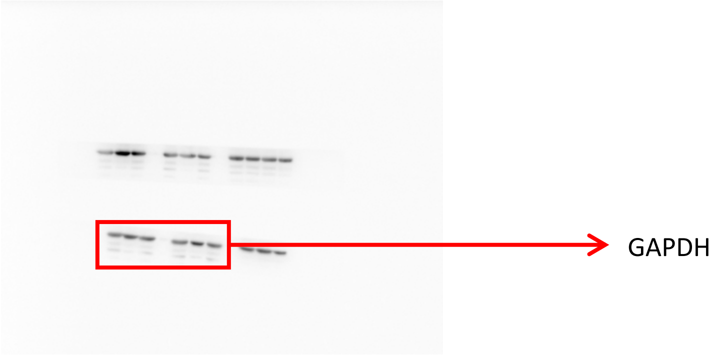

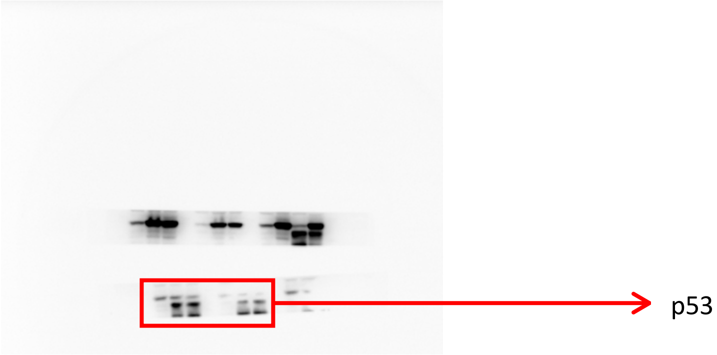

Figure 2A

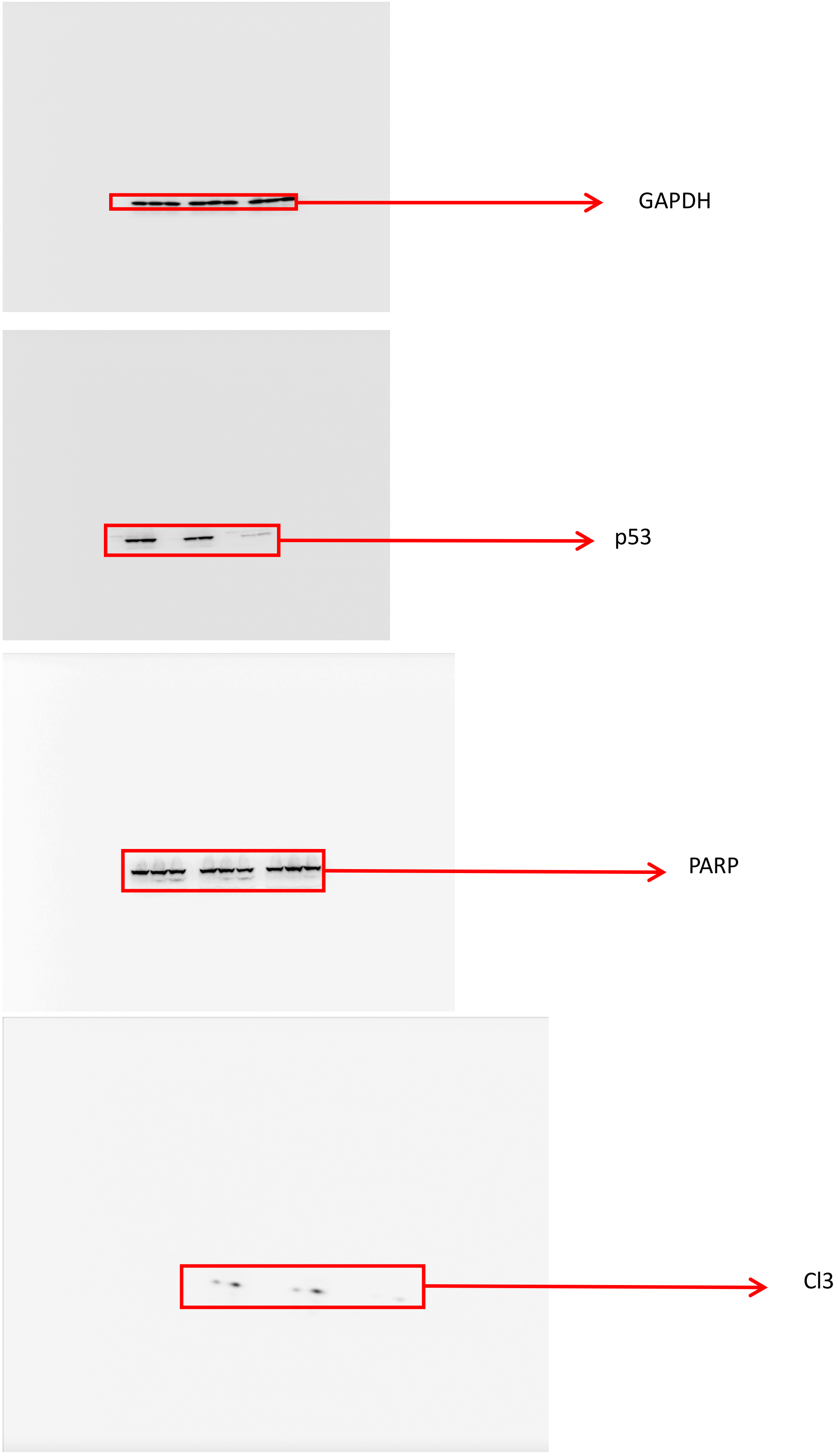

Figure 2D

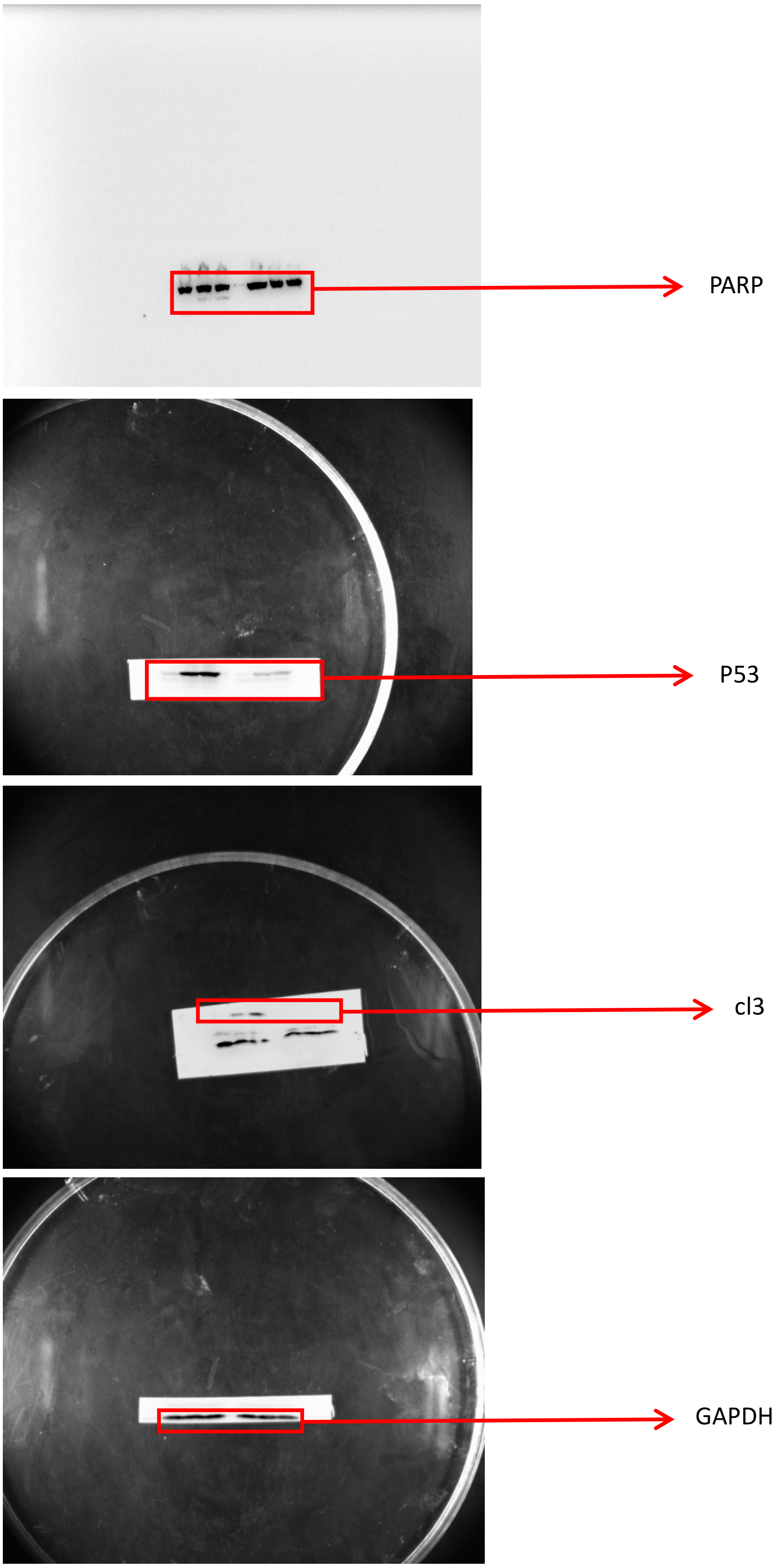

Figure 4A

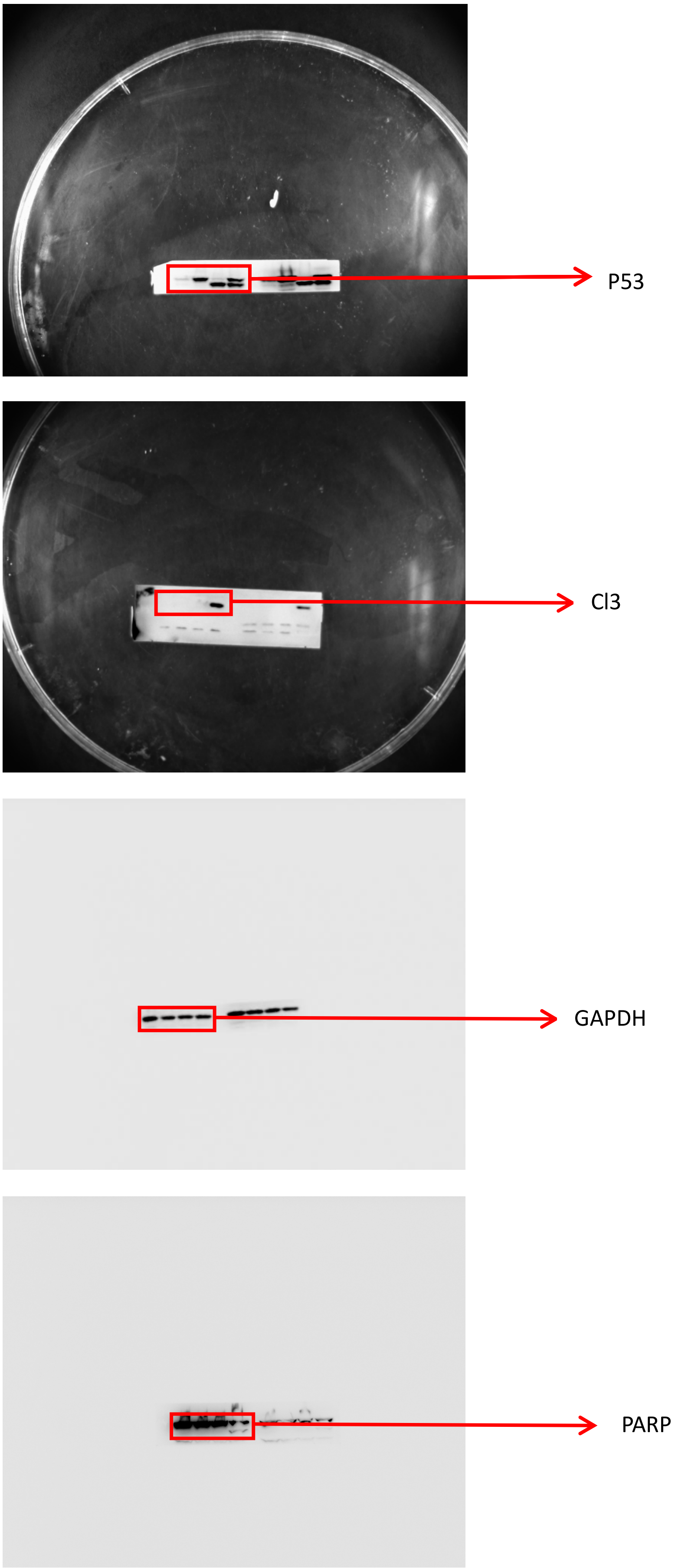

Figure 4B

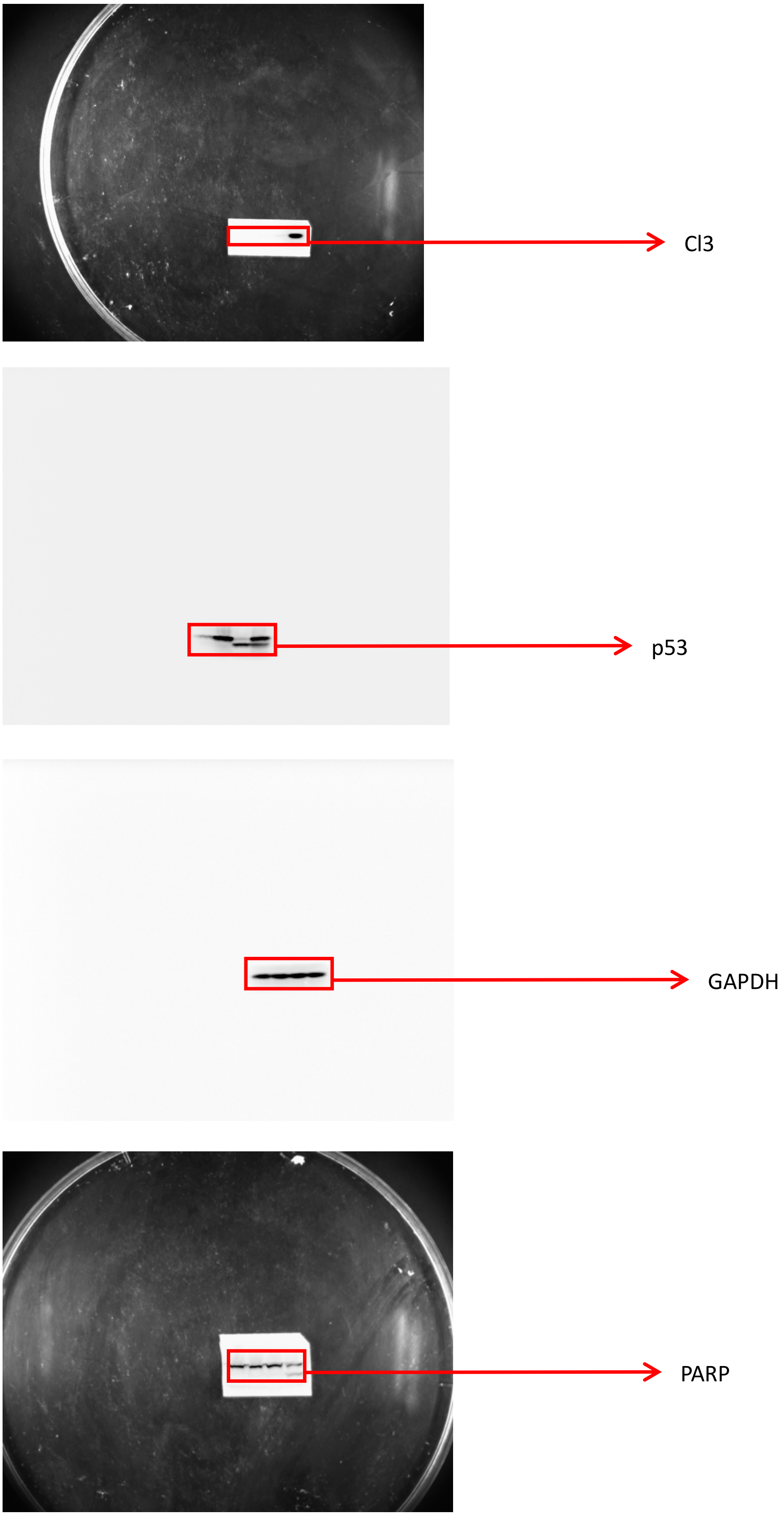

Figure 4C

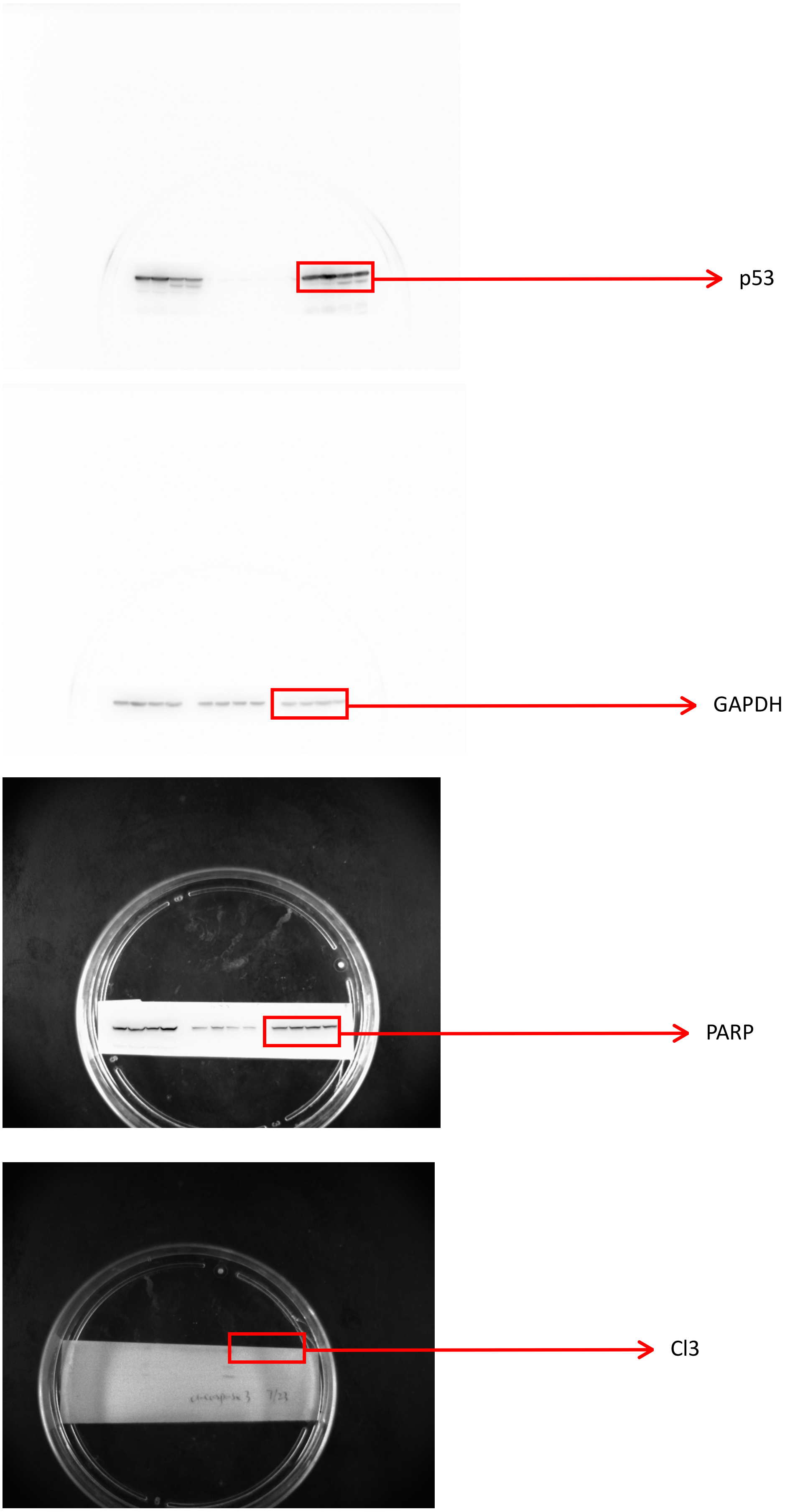

Figure 4D

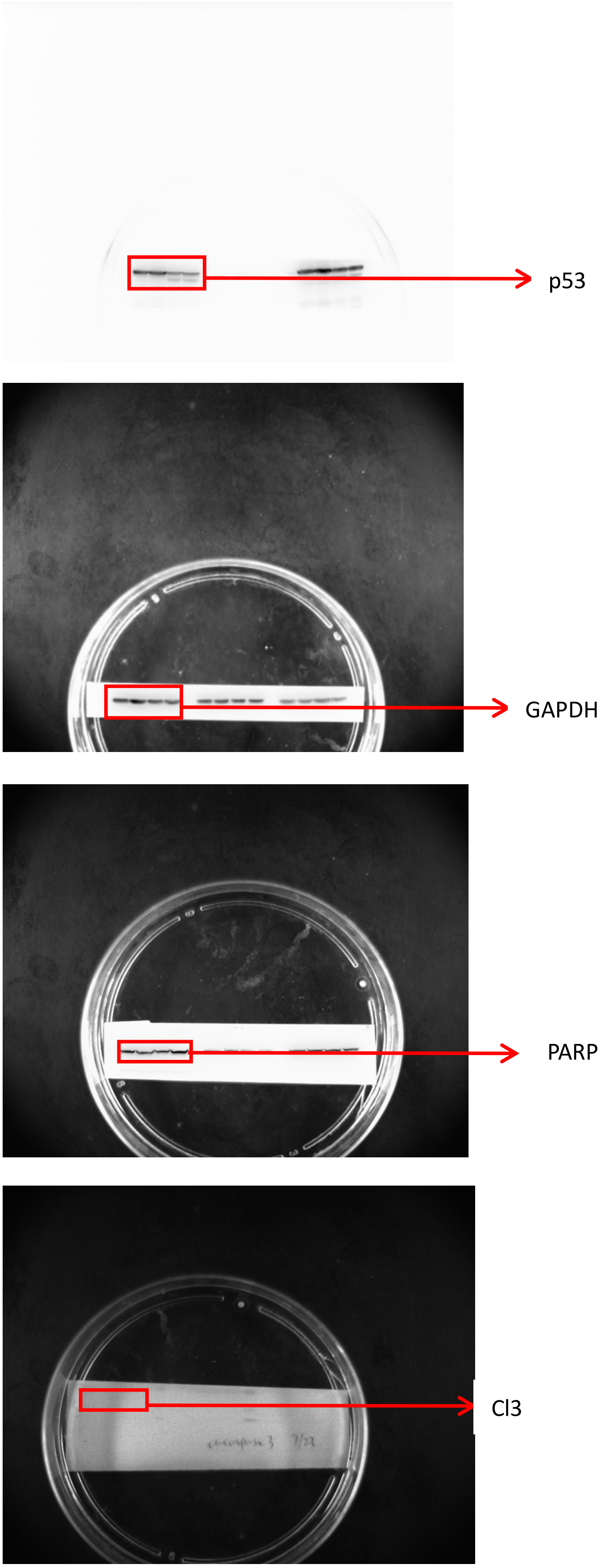

Figure 4E

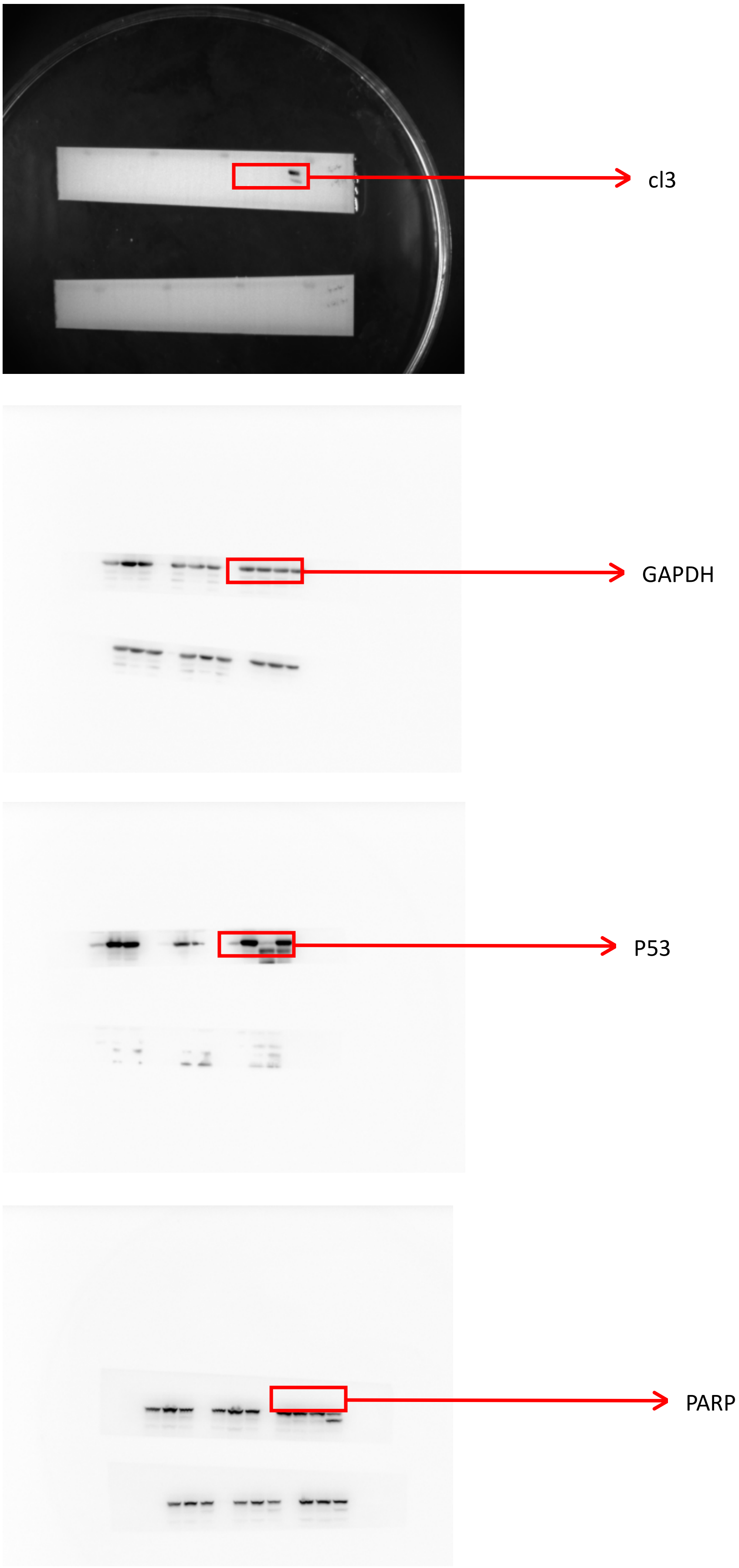

Figure 4F

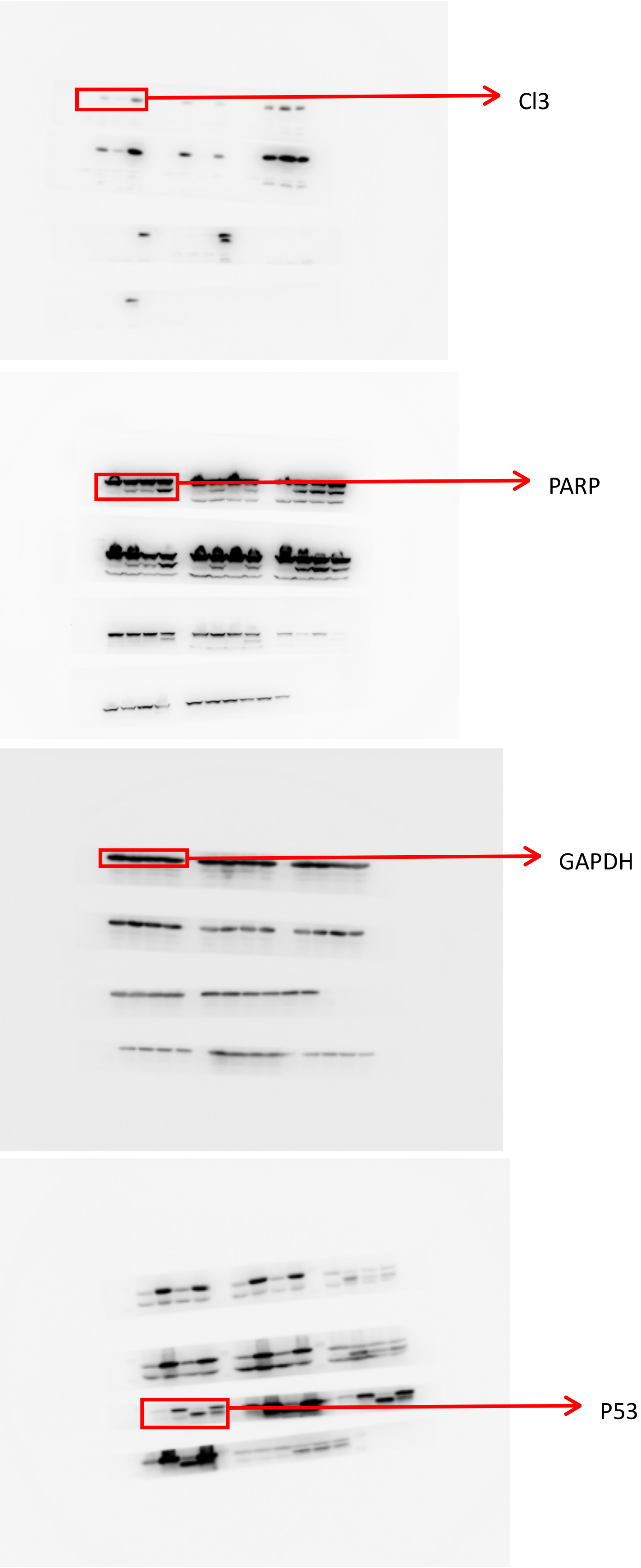

Figure 5A cleaved caspase 3

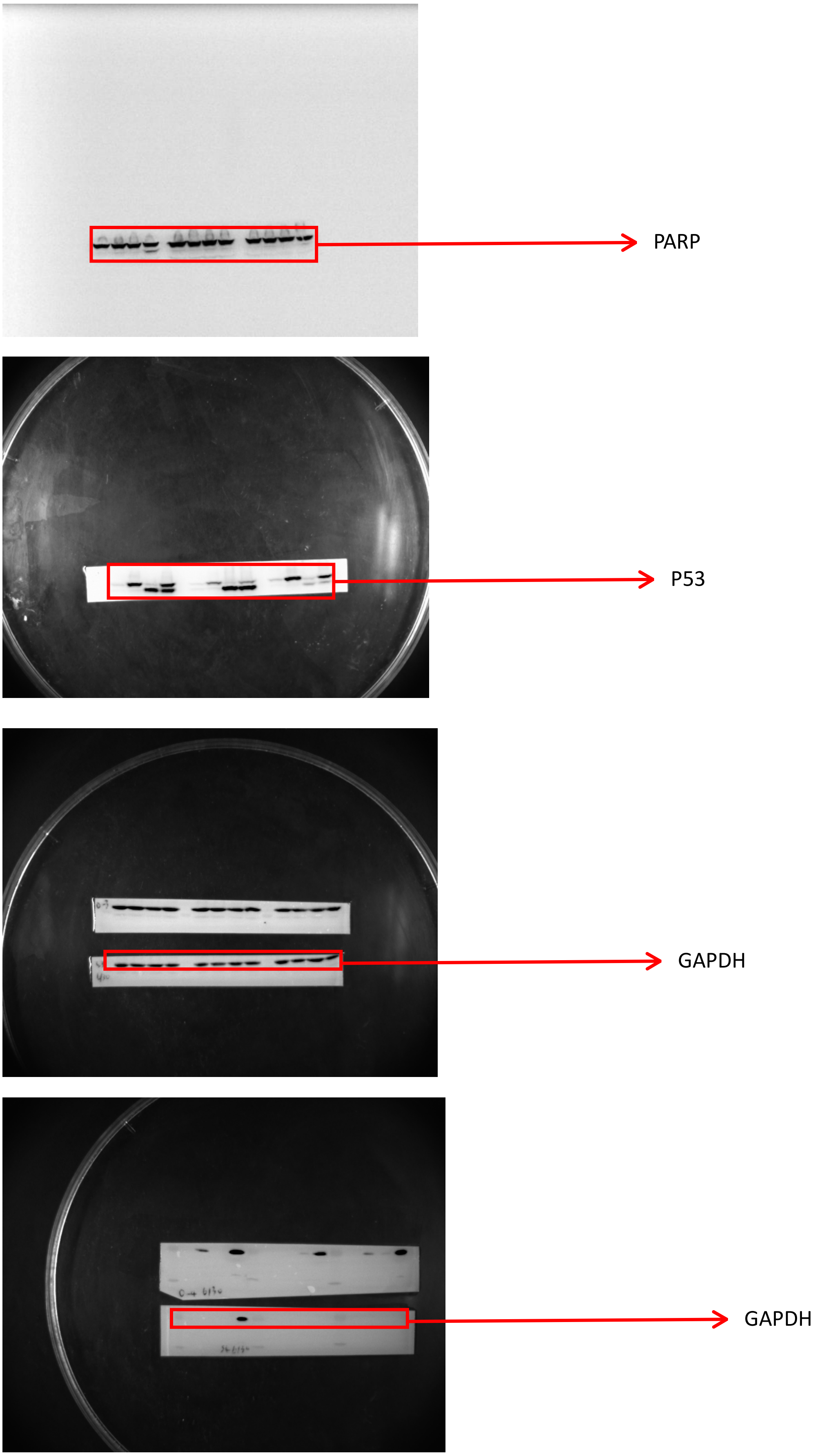

Figure 5B

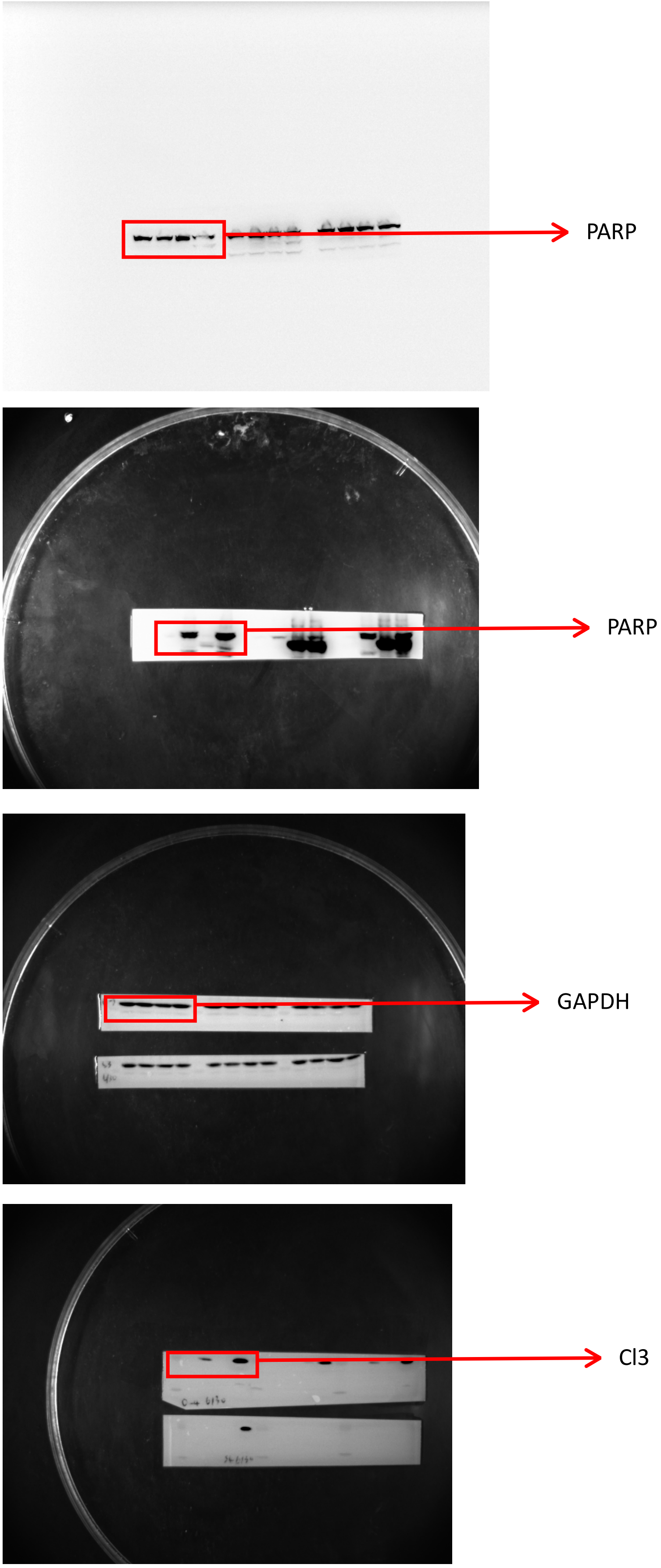

Figure 5C

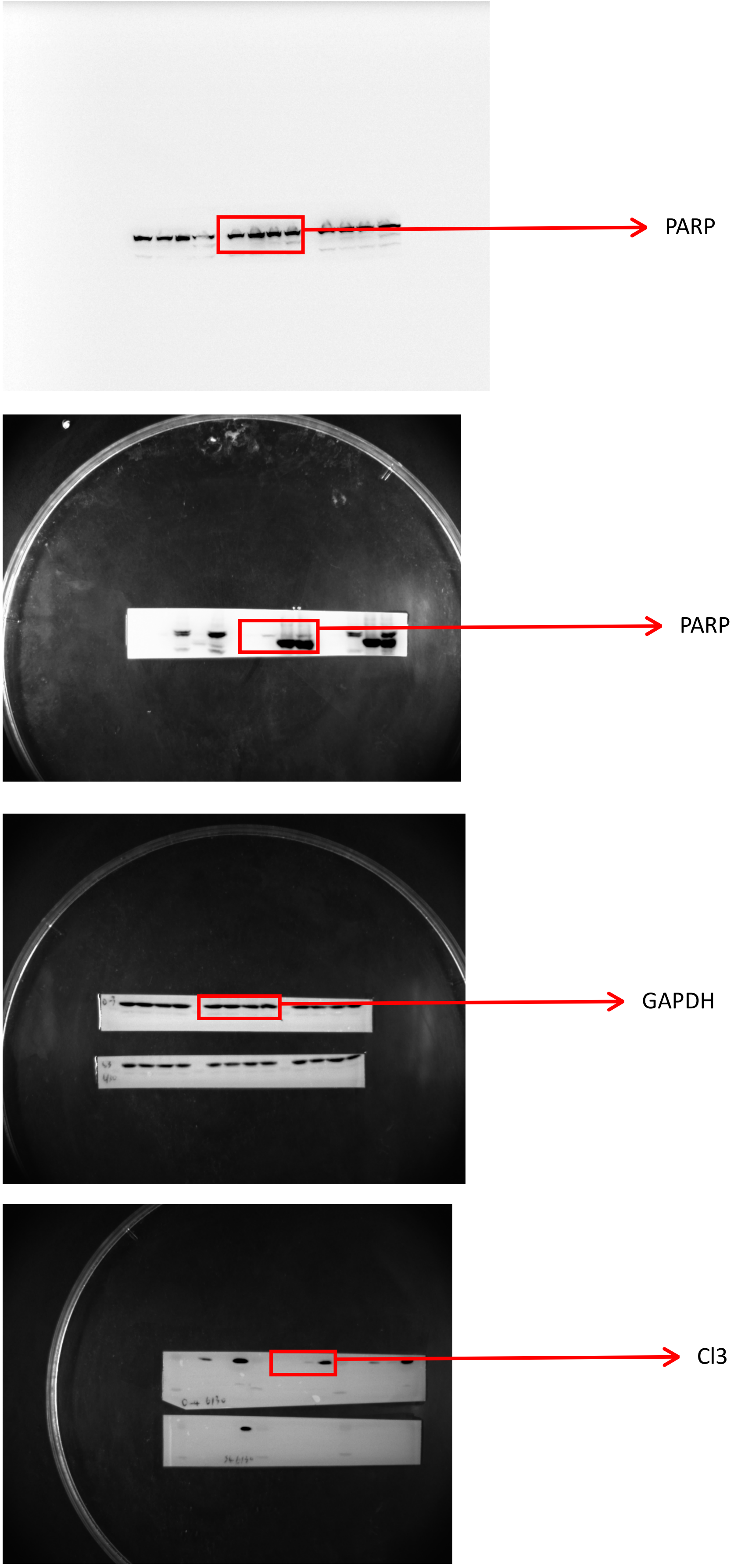

Figure 5D

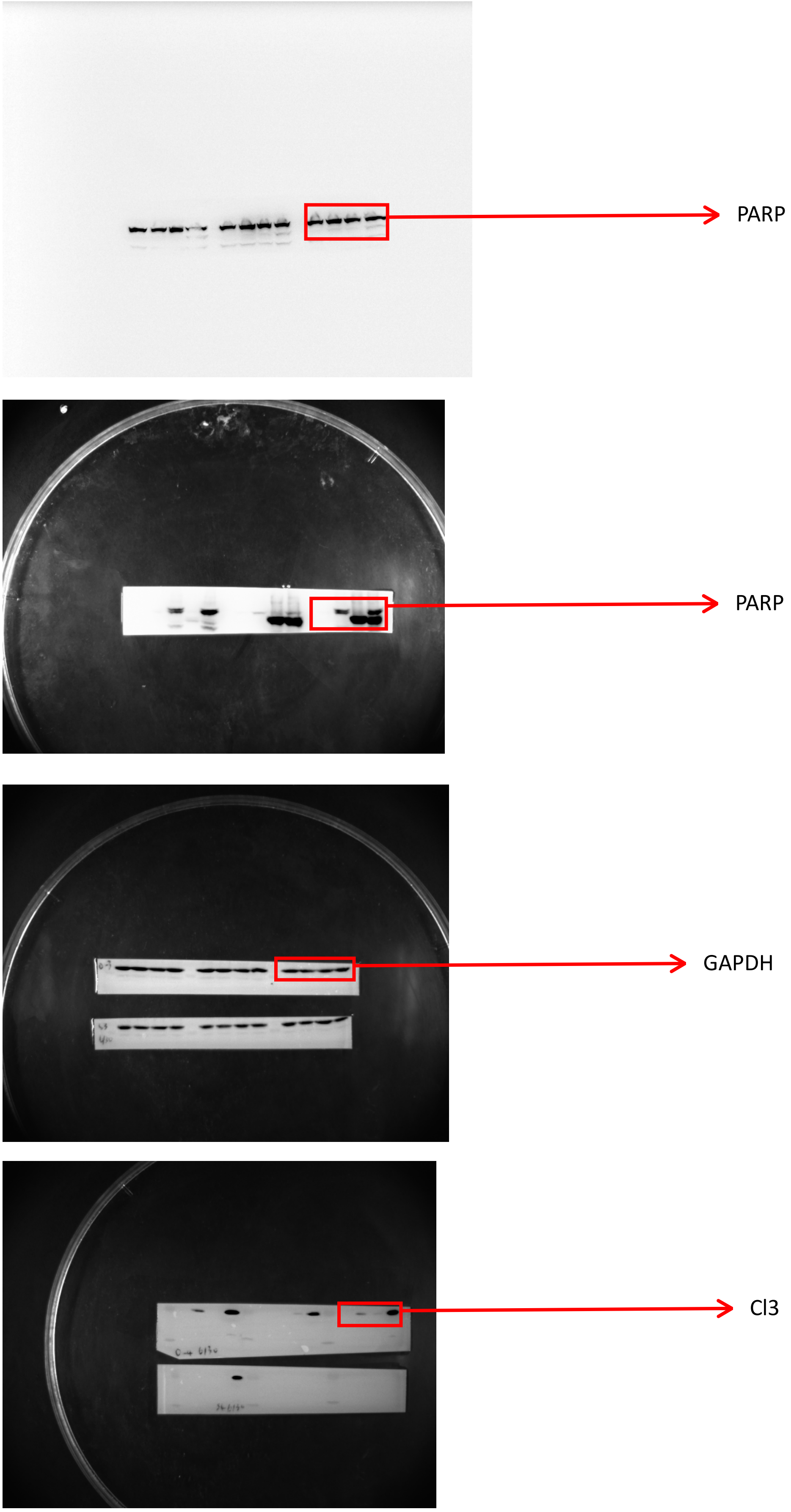

Supplementary Figure 2A

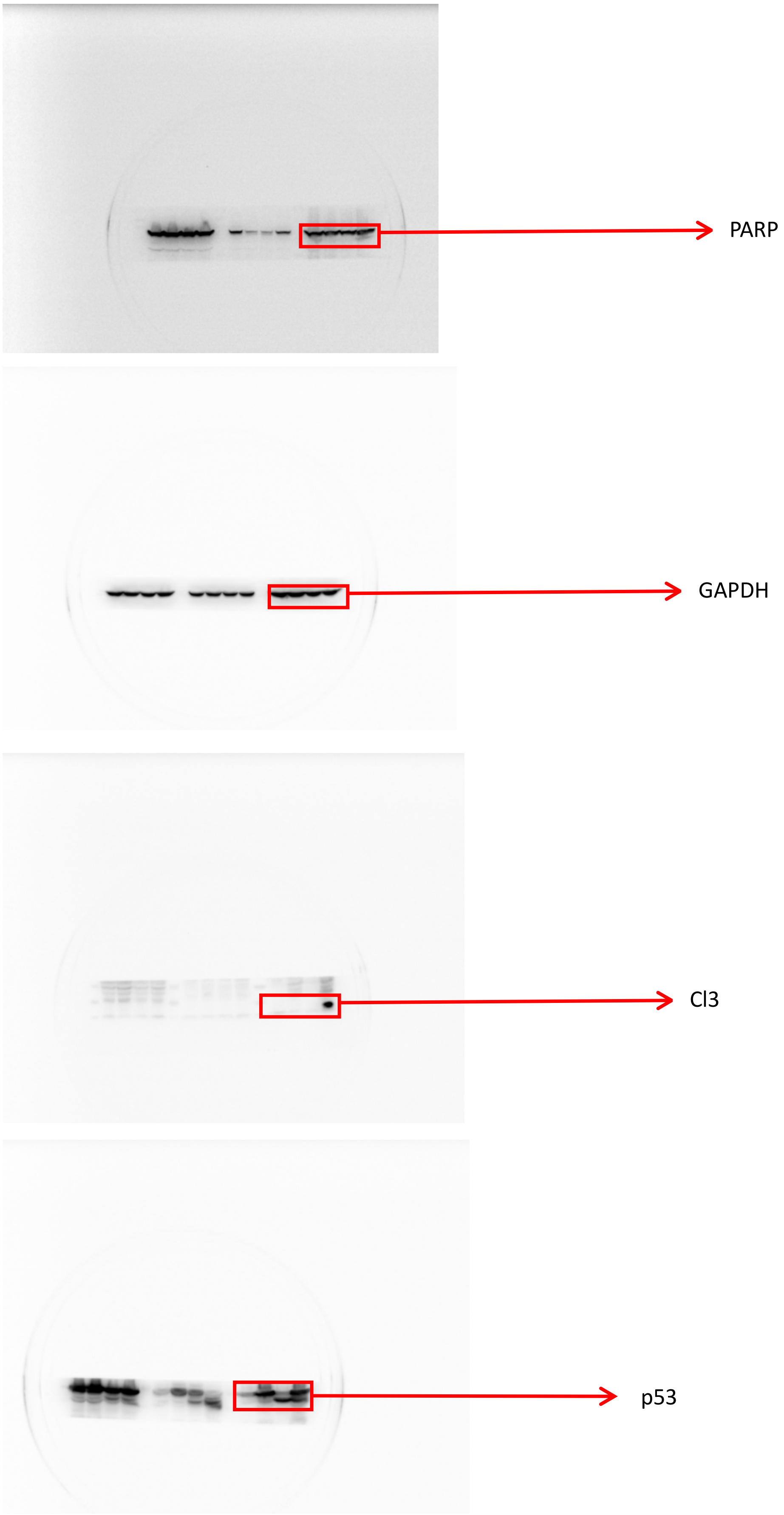

Supplementary Figure 2B

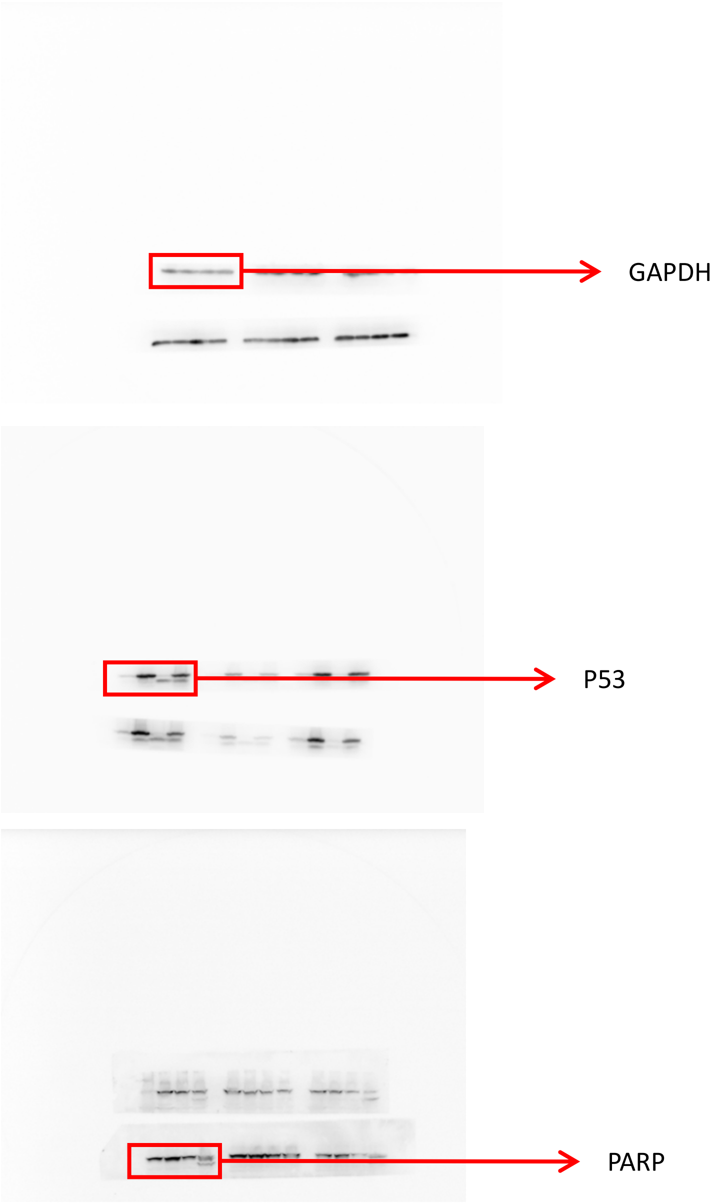

Supplementary Figure 2C

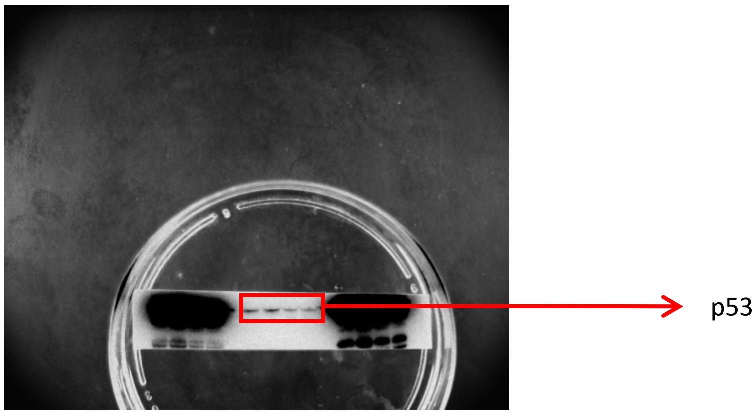

p53

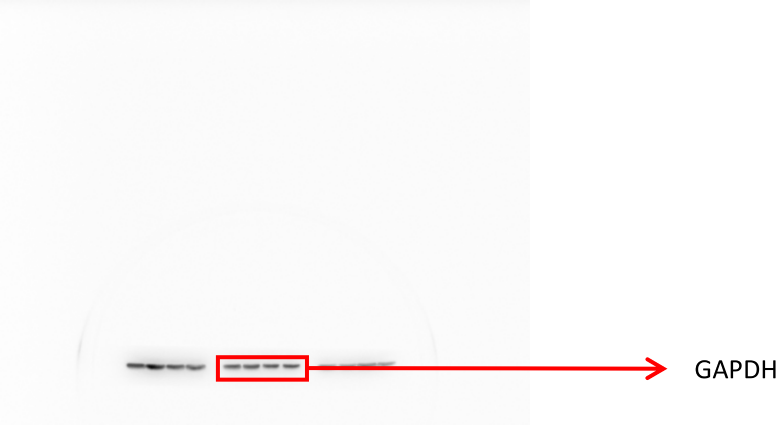

GAPDH

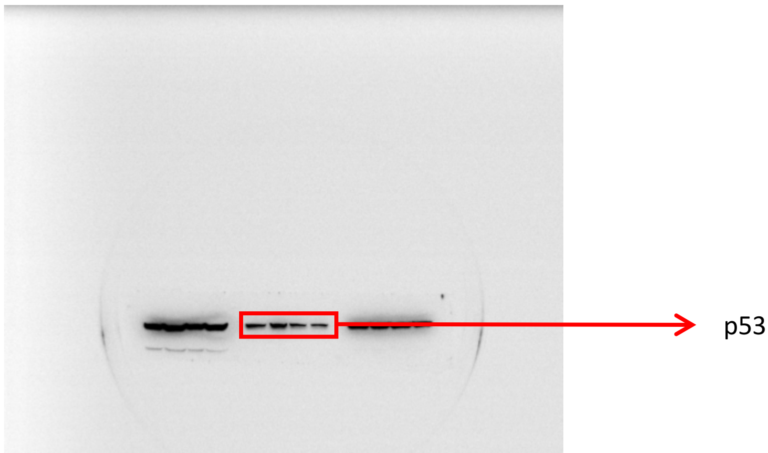

p53

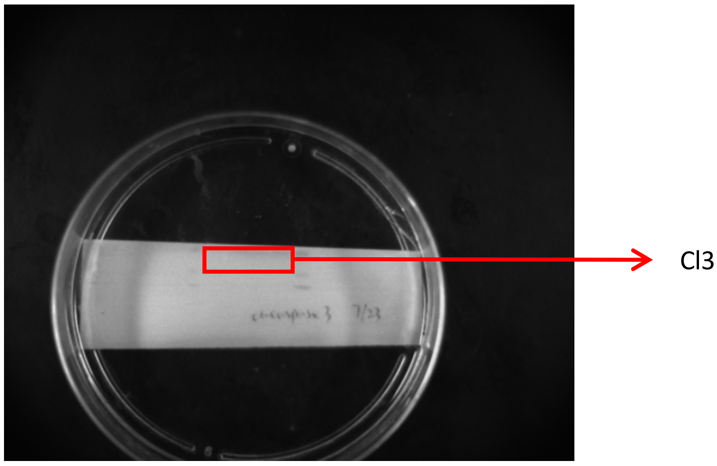

Cl3

Supplementary Figure 2D

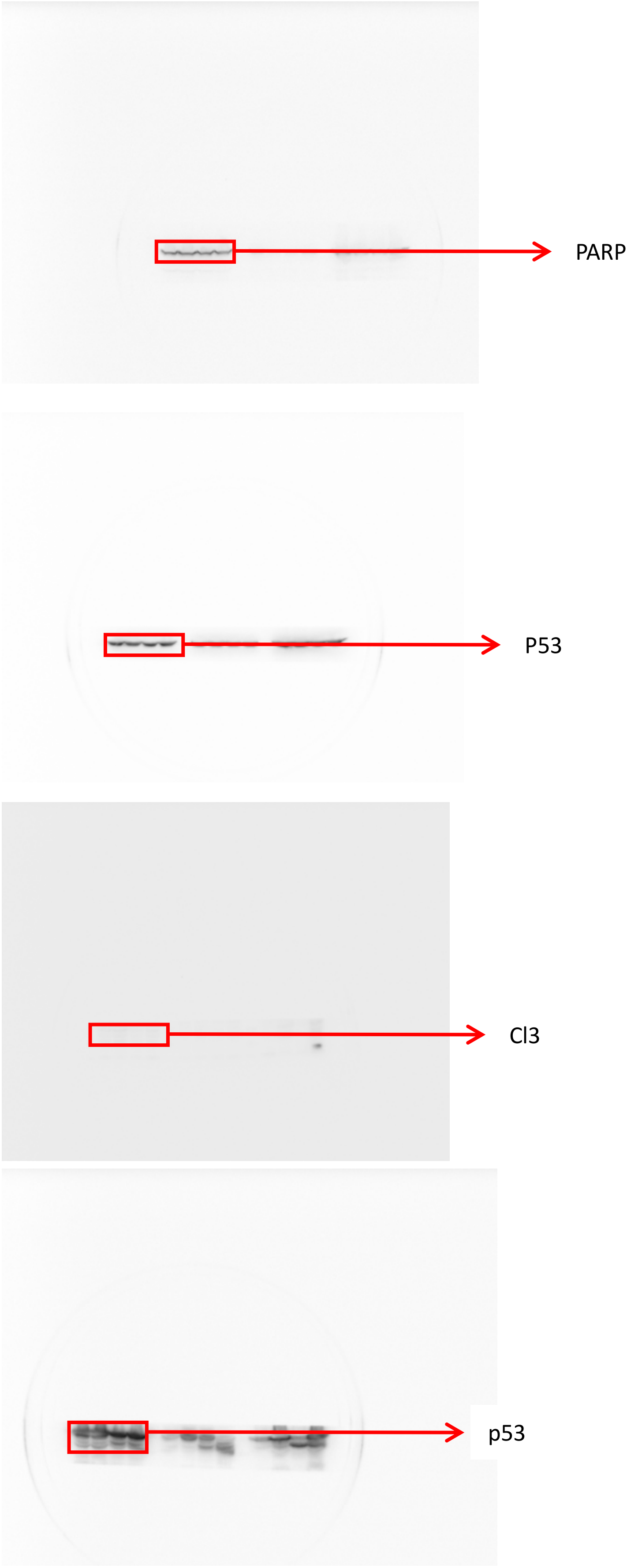

Supplementary Figure 3A

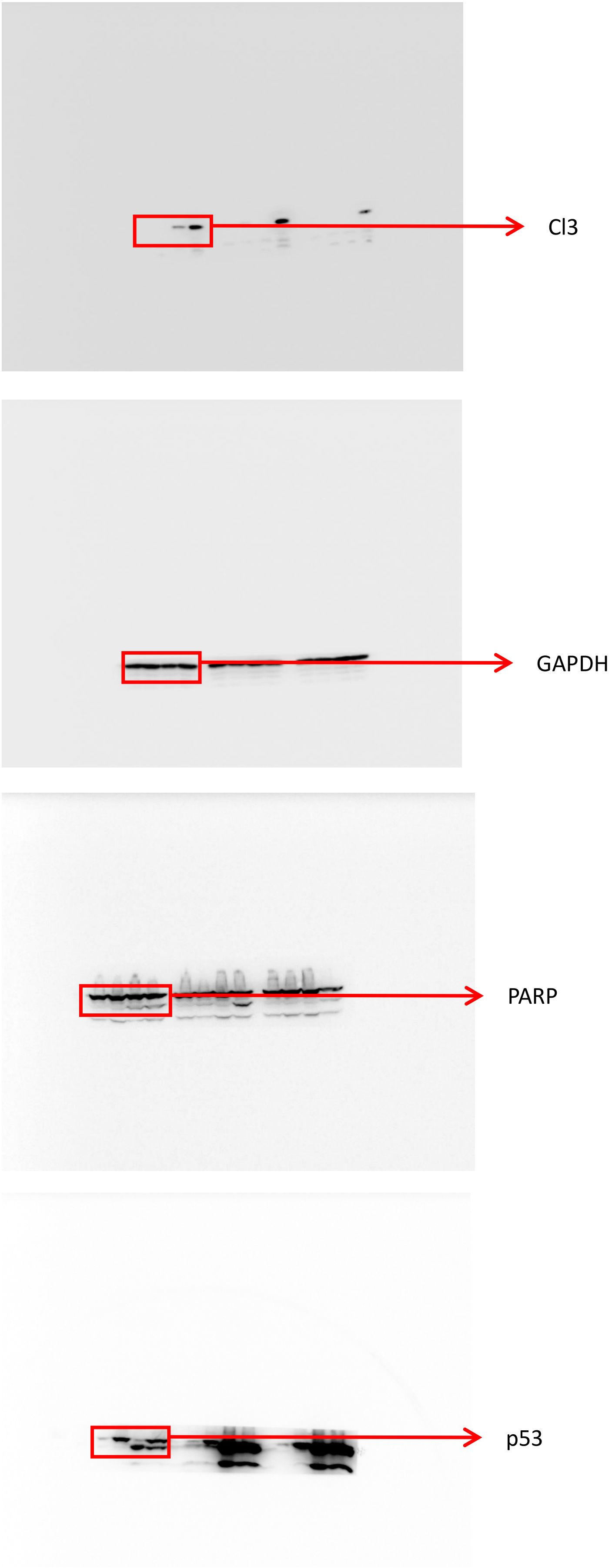

Supplementary Figure 3B

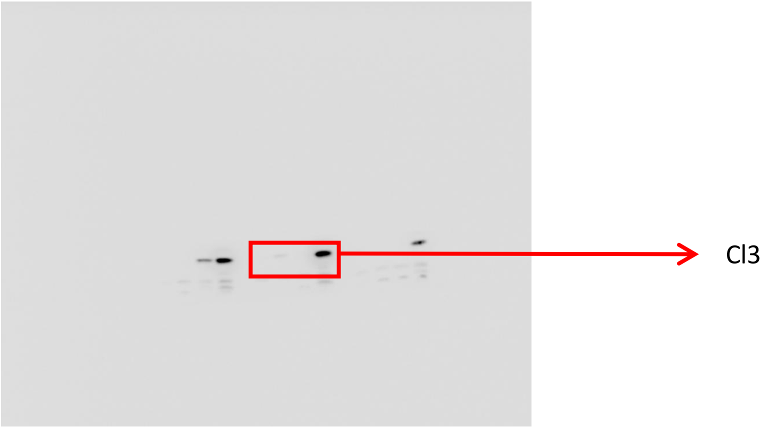

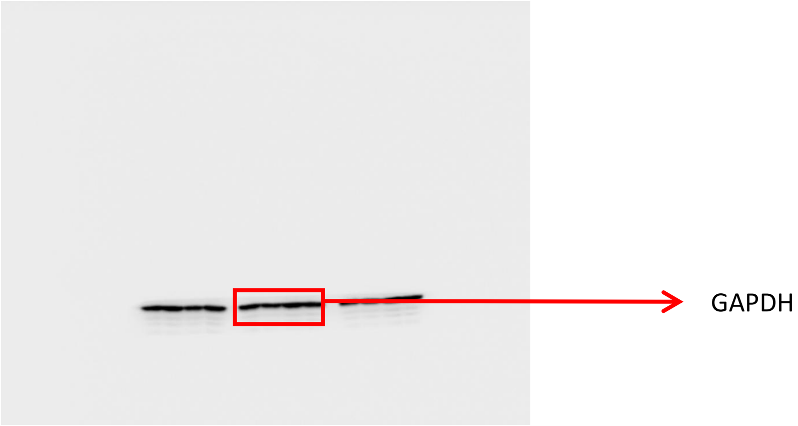

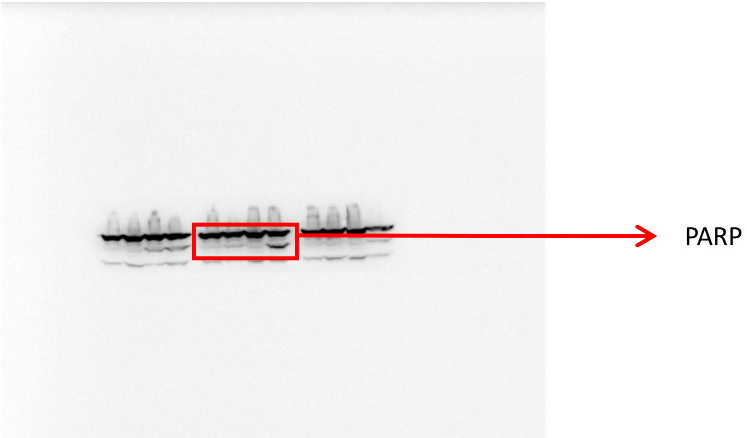

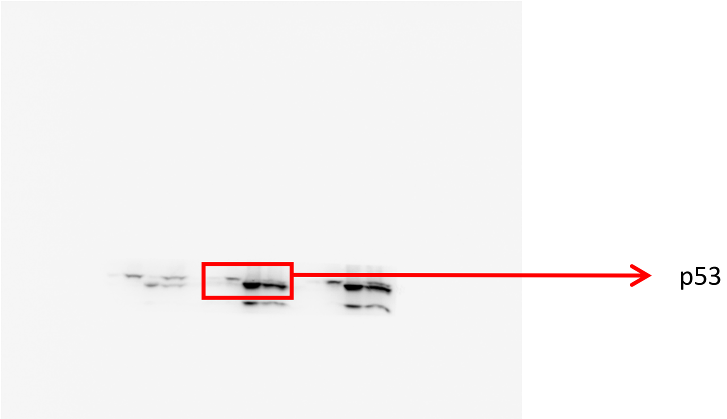

Supplementary Figure 3C

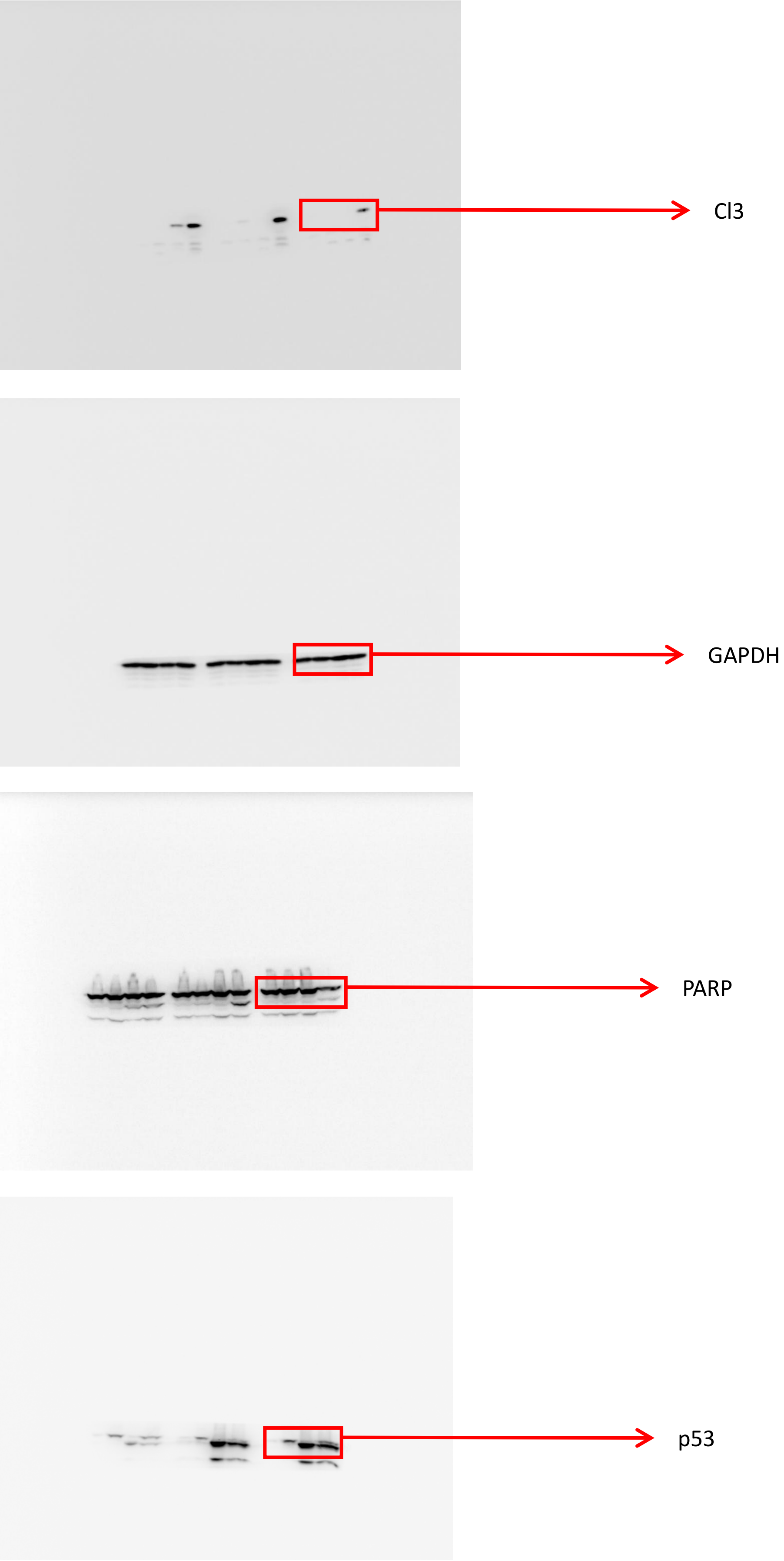

Supplement: Supplementary file 8 — Original Data File [file 41420_2022_1190_MOESM8_ESM.pdf]
